# 2-Cyanopyrimidine-Containing Molecules for N-Terminal Selective Cyclization of Phage-Displayed Peptides

**DOI:** 10.1021/acschembio.4c00725

**Published:** 2025-01-07

**Authors:** J. Trae Hampton, Connor R. Dobie, Demonta D. Coleman, Moulay I. Cherif, Sukant Das, Wenshe Ray Liu

**Affiliations:** † Texas A&M Drug Discovery Center, Department of Chemistry, Texas A&M University, College Station, Texas 77843, United States; ‡ Institute of Biosciences and Technology and Department of Translational Medical Sciences, College of Medicine, Texas A&M University, Houston, Texas 77030, United States; § Department of Biochemistry and Biophysics, Texas A&M University, College Station, Texas 77843, United States; ∥ Department of Cell Biology and Genetics, College of Medicine, Texas A&M University, College Station, Texas 77843, United States

## Abstract

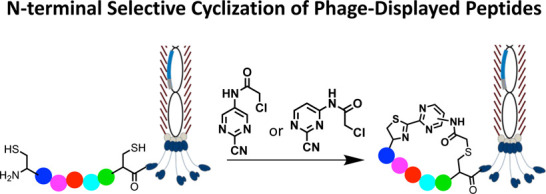

Current methods for the macrocyclization of phage-displayed peptides often rely on small molecule linkers that nonspecifically react with targeted amino acid residues. To expand tool kits for more regioselective macrocyclization of phage-displayed peptides, this study explores the unique condensation reaction between an N-terminal cysteine and nitrile along with the reactivity of an internal cysteine. Five 2-cyanopyrimidine derivatives were synthesized for this purpose and evaluated for their selective macrocyclization of a protein-fused model peptide. Among these, two novel linkers, 2-chloro-*N*-(2-cyanopyrimidin-5-yl)acetamide (pCAmCP) and 2-chloro-*N*-(2-cyanopyrimidin-4-yl)acetamide (mCAmCP), emerged as efficient molecules and were demonstrated to macrocyclize phage-displayed peptide libraries flanked by an N-terminal and an internal cysteine. Using these linkers to generate macrocyclic peptide libraries displayed on phages, peptide ligands for the ZNRF3 extracellular domain were successfully identified. One of the identified peptides, Z27S1, exhibited potent binding to ZNRF3 with a *K*_D_ value of 360 nM. Notably, the selection results revealed distinct peptide enrichment patterns depending on whether mCAmCP or pCAmCP was used, underscoring the significant impact of linker choice on macrocyclic peptide identification. Overall, this study validates the development of two novel regioselective, small molecule linkers for phage display of macrocyclic peptides and highlights the benefits of employing multiple linkers during phage selections.

## Introduction

**Figure 1:**
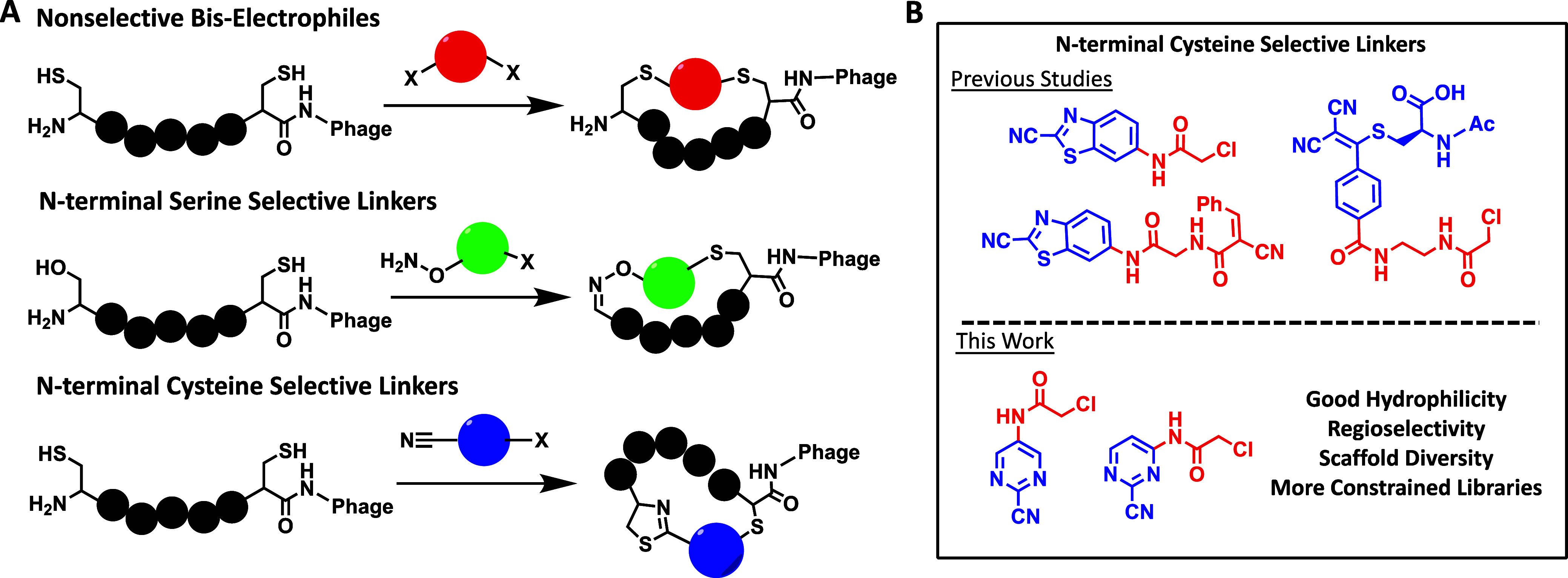
Overview of approaches for phage display of macrocyclic peptides. (A) Initially developed nonselective linkers reacted with two cysteines via activated bis-electrophiles. Two subsequent N-terminal serine and cysteine approaches have been developed that react via hydroxylamines and nitriles to form stable iminoxy and thiazolidine adducts, respectively. (B) N-terminal cysteine selective bis-electrophiles are colored according to reactivity with N-terminal cysteine (blue) or internal cysteine (red). Currently available N-terminal cysteine linkers are limited by either low hydrophilicity or highly flexible scaffolds. The novel linkers reported in this paper give additional diversity of highly constrained scaffolds with good hydrophilicity and regioselectivity.

Until recently, pharmaceuticals have primarily consisted of small molecules (<500 Da) or large biologics (>20 kDa). However, in the past decade, peptides have emerged as promising alternative therapeutic candidates that may combine the stability and ease of manufacturing of small molecules with the specificity of antibody therapeutics. So far, much interest in peptide drugs has centered on creating analogues of natural peptide hormones, such as the recently developed GLP-1 agonists. Nevertheless, novel peptide screening techniques have given the ability to quickly identify peptides that potently bind to desired therapeutic targets.^1−4^ Of the available peptide screening techniques, phage display has been used to generate libraries of up to 10^11^ peptides that can be screened in a variety of conditions without compromising library integrity. However, peptides that are generated during phage production are linear, which typically have limited therapeutic potential because of poor pharmacokinetics.^5^ To counteract this, several different methods have been explored to generate macrocyclic peptides displayed on phage, which exhibit much higher stability toward proteolytic cleavage and improved binding potential due to their constrained structure.^6^ Most reported methods for cyclization of phage libraries rely on highly reactive bis-electrophiles that employ nucleophilic amino acids, typically cysteine, owing to its low natural abundance and high nucleophilicity (Figure 1A).^7,8^ All of these, however, are nonselective, resulting in toxicity to phages by modifying phage coat proteins, which lowers the phage yields. Some reports using genetic code expansion to generate macrocyclic peptides prevent the use of exotic organic linkers by relying on natural biological machinery, but also give low phage expression yields in comparison to libraries containing only canonical amino acids.^9,10^ A promising alternative strategy that has been recently developed is through taking advantage of N-terminal selective reactions to cyclize peptide libraries containing an N-terminal serine or cysteine (Figure 1A).^11−14^ Because these methods use a site-selective reaction with an N-terminal residue, they avoid potential side reactions that modify other phage proteins, minimizing interference with phage proliferation and making them much more compatible with phage display. Despite their increased compatibility with phage display, there are still relatively few examples of linkers that display preferential reactivity toward an N-terminal residue.

While the previously developed N-terminal selective linkers do not result in toxicity to phages, they still have properties that can be optimized. All of the serine-based linkers rely on oxidation of the N-terminus to an aldehyde using sodium periodate, followed by a buffer exchange step.^11^ On the other hand, the N-terminal cysteine linkers are simpler, as they can be reacted with phages in one pot under specific conditions.^12−14^ Of the cysteine-reactive linkers, all take advantage of electron-deficient nitriles that selectively condense with 1,2-aminothiols to produce stable thiazolidine adducts (Figure 1B). While this affords high reactivity toward the N-terminus, it relies on large, complex cores to activate the nitrile that can significantly affect peptide selections. Also, the hydrophobicity of the cyanobenzothiazole linkers limits their potential in phage selections, as they can hinder solubility of the identified peptides and may even prevent them from interacting well with proteins.^15,16^ In this work, we report two novel hydrophilic linkers that react selectively with the N-terminal cysteine and demonstrate these linkers in the identification of macrocyclic peptides for ZNRF3, a ubiquitin E3 ligase that shows promise in developing proteolysis-targeting chimeras for membrane proteins.

## Results

### Identification of 2-Cyanopyrimidines for N-Terminal Selective Peptide Macrocyclization

**Figure 2:**
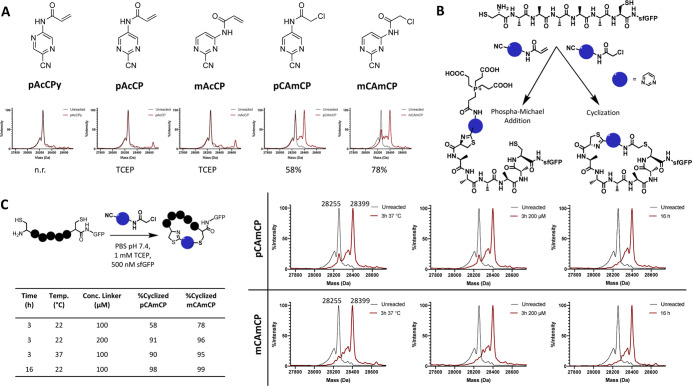
Identification of modified 2-cyanopyrimidines for the cyclization of an N-terminal cysteine-containing peptide fused to the N-terminus of sfGFP. (A) Five different potential N-terminal selective bis-electrophiles were screened for reactivity with the CA_5_C-sfGFP protein and analyzed via LC-ESI-MS after reacting for 3 h at RT. Percent yields were calculated by the ratio between desired product and remaining unreacted protein. N.r. = no observed reaction; TCEP = the only observed adducts included a TCEP modification of acrylamide. (B) Two possible reaction paths were observed for the linkers dependent on the secondary electrophile being an acrylamide or 2-chloroacetamide moiety. Linkers containing acrylamides reacted with TCEP via a phospha-Michael addition, while the 2-chloroacetamide compounds gave the desired cyclic product. (C) CAmCP linkers were optimized for their reaction with CA_5_C-sfGFP. The percent cyclized under each condition was calculated by the ratio of the peaks in the deconvoluted mass spectra (right). Masses corresponding to the unreacted product (28,255 Da) and cyclized product (28,399 Da) are labeled.

In our search for desirable scaffolds to macrocyclize phage-displayed peptide libraries containing an N-terminal cysteine and an internal cysteine, we looked to take advantage of electron-deficient nitriles that can selectively react with the N-terminal cysteine and ultimately result in a stable thiazolidine adduct. Similar to the previously identified 2-cyanobenzothiazoles that display extremely high reactivity with the N-terminal cysteine,^17,18^ we envisioned that other nitrogen-rich heterocycles would be electron withdrawing enough to activate nitriles for the condensation reaction between the nitrile group and N-terminal cysteine. Previous studies have predicted 2-cyanopyrimidines and 2-cyanopyrazines to contain highly activated nitriles.^19,20^ Thus, we envisioned that 2-cyanopyrimidines and 2-cyanopyrazines would provide substantial reactivity with the N-terminal cysteine to preferentially tune the macrocyclization for phage-displayed peptide libraries. In addition to varying the heterocyclic core, we also looked to understand how the second electrophilic moiety position and structure affected reactivity. To provide selectivity for N-terminal cysteine libraries, the reaction with the internal cysteine should be much slower than the initial N-terminal cysteine condensation. Traditional cysteine-reactive electrophiles that are relatively inert at physiological pH include acrylamides and chloroacetamides, both of which show reaction rates at least 100-fold slower than the CBT condensation reaction.^21,22^ By coupling these slowly reacting electrophiles with quick thiazolidine formation from the nitrile condensation to the N-terminal cysteine, the reaction is kinetically controlled to selectively react with proteins containing an N-terminal cysteine. Therefore, we designed five different molecules that were either 2-cyanopyrimidine or 2-cyanopyrazine fused to chloroacetamide or acrylamide either meta or para to the nitrile group (Figure 2A). These compounds were readily synthesized through simple one step reactions from their commercially available 2-cyano derivatives, purified through chromatographic techniques, and characterized with NMR and high-resolution mass spectrometry (synthetic schemes and characterization data are available in the Supporting Information, Figures S1–S15).

The designed organic linkers were tested for their ability to cyclize a CA_5_C peptide expressed as an N-terminal fusion to sfGFP (CA_5_C-sfGFP). Following reduction of the protein with 1 mM TCEP for 30 min, 100 μM compounds were reacted with the reduced CA_5_C-sfGFP for 3 h in PBS (pH 7.4). Following the reaction, the protein was analyzed via electrospray ionization mass spectrometry (ESI-MS) analysis to determine the desired adduct and any potential side reactions that occurred. The 2-cyanopyrazine (pAcCPy) demonstrated essentially no reactivity with the protein, but all pyrimidine derivatives exhibited reactivity with the N-terminal cysteine (Figure 2A). This is likely due to the electron withdrawing effects of the nitrogen atoms adjacent to the nitrile in the 2-cyanopyrimidine derivatives. Of the pyrimidine derivatives, both compounds containing chloroacetamide demonstrated significantly higher reactivity and specificity in comparison to those species containing acrylamide. The compound containing the 2-chloroacetamide group at the meta position of the pyrimidine with respect to the cyano group (mCAmCP) exhibited nearly 70% cyclization after 3 h, while the para derivative (pCAmCP) showed 50% cyclization. Following previously established Bayesian deconvolution of the mass spectra,^23^ protein masses from each reaction (MW = 28,399 Da) corresponded well with that of the expected cyclic product (MW = 28,399 Da). On the other hand, the acrylamide-containing derivatives (pAcCP and mAcCP) did not result in the expected CA_5_C-sfGFP cyclization. As shown in Figure 2A, we observed an adduct (28,665 Da) that was 251 Da higher than the expected cyclic product (28,414 Da). This observed mass matches a TCEP-conjugated product resulting from a previously reported competing phospha-Michael addition of TCEP to the acrylamide moiety.^24^ This product has a TCEP-functionalized linker that is nonreactive toward the internal cysteine (Figure 2A,B). Following these initial studies, we then further optimized the cyclization of CA_5_C-sfGFP using pCAmCP and mCAmCP by varying the reaction time, temperature, and concentration of linkers (Figure 2C). Ultimately, reacting the protein with 100 μM linker for 16 h provided optimal conditions, resulting in nearly complete cyclization of CA_5_C-sfGFP with no significant side reactions observed (Figure 2C). These results demonstrated the great potential of using pCAmCP and mCAmCP to cyclize peptides on proteins in physiological conditions.

**Figure 3:**
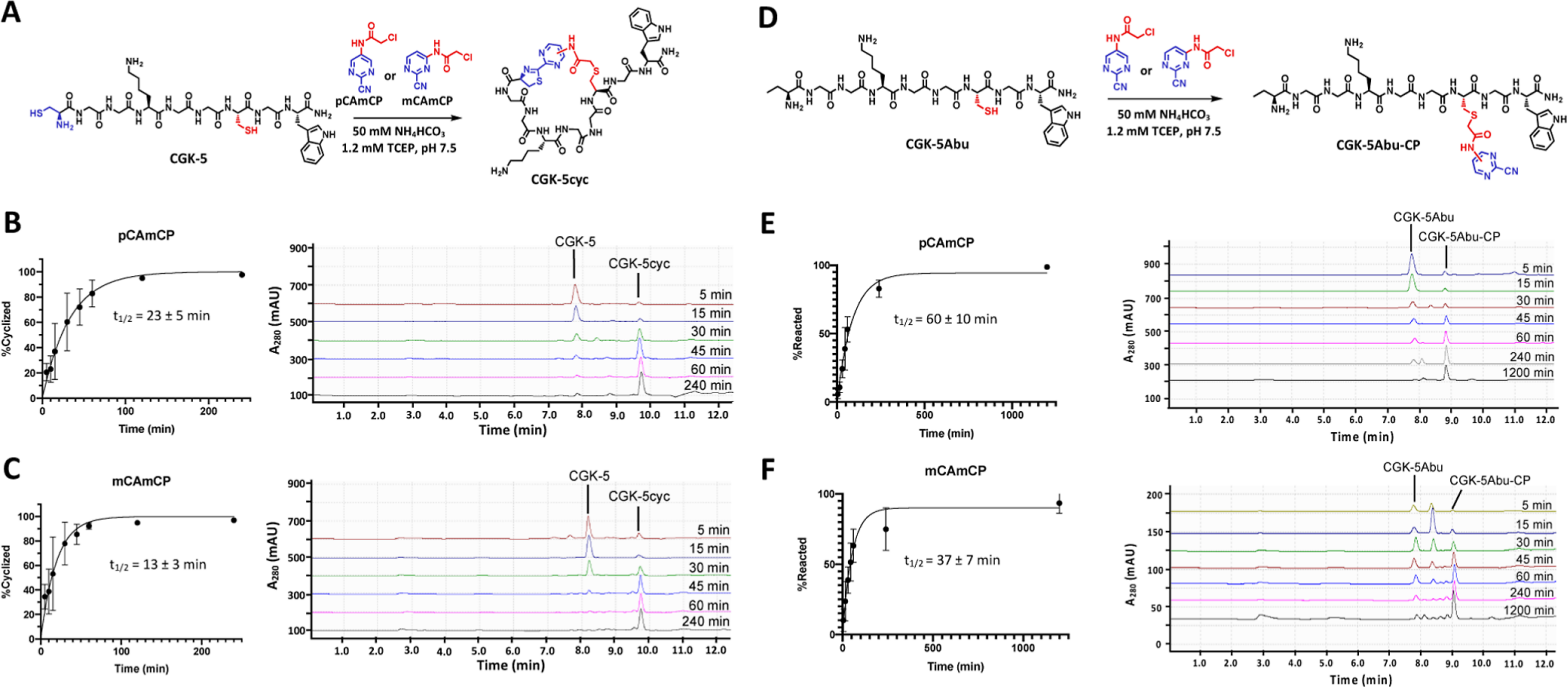
Kinetic characterization of the CAmCP linkers reacting with a model peptide. (A) Peptide CGK-5 was reacted with the CAmCP linkers to afford the cyclized product CGK-5cyc. Representative HPLC traces and integrated peaks for the kinetic experiments with pCAmCP (B) and mCAmCP (C) are shown. (D) For studying the chloroacetamide addition, CGK-5Abu was reacted with the CAmCP linkers. Representative HPLC traces and integrated peaks are shown for the reactions with pCAmCP (E) and mCAmCP (F). Each data point is shown as the mean ± s.d. of at least three independent experiments. Half-lives are reported as the mean ± 95% CI of the fitted curves.

While pCAmCP and mCAmCP gave efficient cyclization of the peptide fusion of sfGFP, we looked to further investigate the specificity of the reaction using peptide kinetic assays. To do this, we first established reactivity of the compounds with a model peptide (CGK-5, Figures 3A and S16) that was composed of two cysteines flanking a region tuned for solubility in buffer (GGKGG) and with a tryptophan residue on the C-terminus for UV absorbance. Using 500 μM peptide and 550 μM linker in ABC buffer (pH 7.4) containing 1 mM TCEP, LC–MS analysis demonstrated nearly complete cyclization of the peptide after 2 h of reaction at RT, with the half-lives for the reactions being 23 ± 5 and 13 ± 3 min for pCAmCP and mCAmCP, respectively (Figure 3B,C). To test the specificity of the reaction for the N-terminal cysteine, we synthesized a peptide analogue (CGK-5Abu, Figures 3D–F and S17) that contained 2-aminobutyric acid in place of the N-terminal cysteine. While we observed the chloroacetamide addition to the internal cysteine when using 500 μM of this peptide, reaction occurred at a significantly slower rate (∼3-fold slower) in comparison to the cyclization reaction (Figure 3E,F). Since these reactions occurred at 500 μM of peptide to allow for analysis on the HPLC, we envision this difference will likely be exaggerated when using lower peptide/protein concentrations. Although it is unlikely at neutral pH, the chloroacetamide moiety can possibly react with nucleophilic amino acids other than cysteine. To defuse this concern, we designed a peptide containing a variety of possibly nucleophilic residues (serine, arginine, tyrosine, histidine, and lysine, but not an internal cysteine) adjacent to the N-terminal cysteine (BCP, Figure S18) and let the peptide react with pCAmCP or mCAmCP. After monitoring the reaction over 4 h, the chloroacetamide was left intact, with the only observed side reaction being the addition of TCEP to the chloroacetamide (Figure S19). On the other hand, peptides that contained an internal cysteine were nearly completely reacted with the chloroacetamide after less than 2 h at the same concentrations (Figure 3B,C). This indicates high specificity for the chloroacetamide reacting with an internal cysteine in comparison to other nucleophilic residues at pH 7.4. This result also corroborates the CA_5_C-sfGFP cyclization data, where no nonselective chloroacetamide adducts to additional amino acid residues within the protein were observed after 16 h of reaction (Figure 2C). Previous studies have also demonstrated the competition between nucleophiles and TCEP toward haloacetamides, particularly iodoacetamides.^25^ It could be that the side reaction with TCEP may help mask the chloroacetamide after long incubation periods to prevent it from reacting with less reactive nucleophiles. These data further validated pCAmCP and mCAmCP as efficient organic linkers for peptides containing both N-terminal and internal cysteine residues.

### Validation of 2-Cyanopyrimidines Functionalized with 2-Chloroacetamide for Phage Display

**Figure 4:**
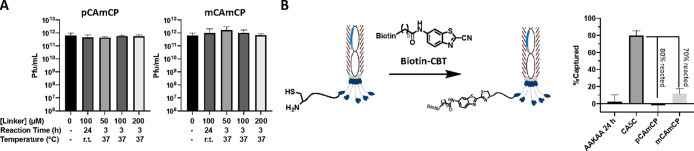
Validation of linkers pCAmCP and mCAmCP for phage display. (A) No observed phage toxicity occurred for either linker when reacted with up to 200 μM of compound and at varying reaction temperatures. Pfu/mL = total infectious phage units per mL quantified via colony-forming unit assays. All assays are reported as mean ± s.d. for *n* = 3 replicates. (B) Biotin pulldown assays indicated high reactivity of the CAmCP linkers with phages that contain N-terminal cysteines. Phages were quantified for their ability to be captured on streptavidin beads using Biotin-CBT to modify free N-terminal cysteines. After reaction with CAmCP linkers, a CA_5_C-modified phage library had no significant differences compared to an unmodified AAKAA library. % Captured calculated as (phages input – phages in supernatant)/phages input. All experiments are reported as mean ± s.d. for *n* = 3 replicates.

Following the characterization of the linkers for efficient cyclization of peptides, we then looked to characterize pCAmCP and mCAmCP for reactivity toward phage proteins. Some previously developed linkers that generate macrocyclic peptide libraries on phage hinder phage infectivity, which is likely from modifying cysteines within phage coat proteins.^26−28^ To test if this occurred in the novel linkers, we performed toxicity assays that reacted the phages with different concentrations of linker (50–200 μM), temperatures (22 or 37 °C), and reaction times (3 or 24 h). No toxicity was observed under any of the conditions tested, indicating that there were no adverse reactions occurring even when incubating phages with extended reaction times (Figure 4A). This is similar to previous reports of other N-terminal cysteine selective linkers that exhibit essentially no toxicity to phage proteins.^12−14^ Following verification that the reaction conditions were not toxic to phages, we then looked to use biotin pulldown assays to confirm reactivity with N-terminal cysteines displayed on the pIII phage protein. We expressed phages with a CA_5_C peptide on the N-terminus of pIII. These phages were subsequently reduced using 1 mM TCEP and then reacted with 100 μM linkers (pCAmCP or mCAmCP) for 24 h at RT. Following the precipitation of the phages, they were again reduced with TCEP and were incubated with a Biotin-CBT compound to label any unreacted N-terminal cysteines with biotin, allowing for phage capture on streptavidin beads (Figure 4B). After reactions with either pCAmCP or mCAmCP, the percent of phages captured was reduced to levels similar to those of a control phage that lacked N-terminal cysteines for capture (AAKAA), thus indicating successful modification of the N-terminus (Figure 4B). Comparison with capture levels of unreacted phages indicated at least 80% and 70% reactivity of pCAmCP and mCAmCP with the CA_5_C phages, respectively. As only 80% of the phages were captured initially using the Biotin-CBT compound, this was the detection limit of the assay, so cyclization efficiencies may even be higher than the reported activities. These efficiencies are also similar to other reported linkers for phage display that have been previously validated through numerous selections.^13,14,29−31^ From these biotin pull-down assays, we thus concluded that both pCAmCP and mCAmCP were able to selectively react with phages containing N-terminal cysteines.

### Selection of Macrocyclic Peptides for ZNRF3

**Figure 5:**
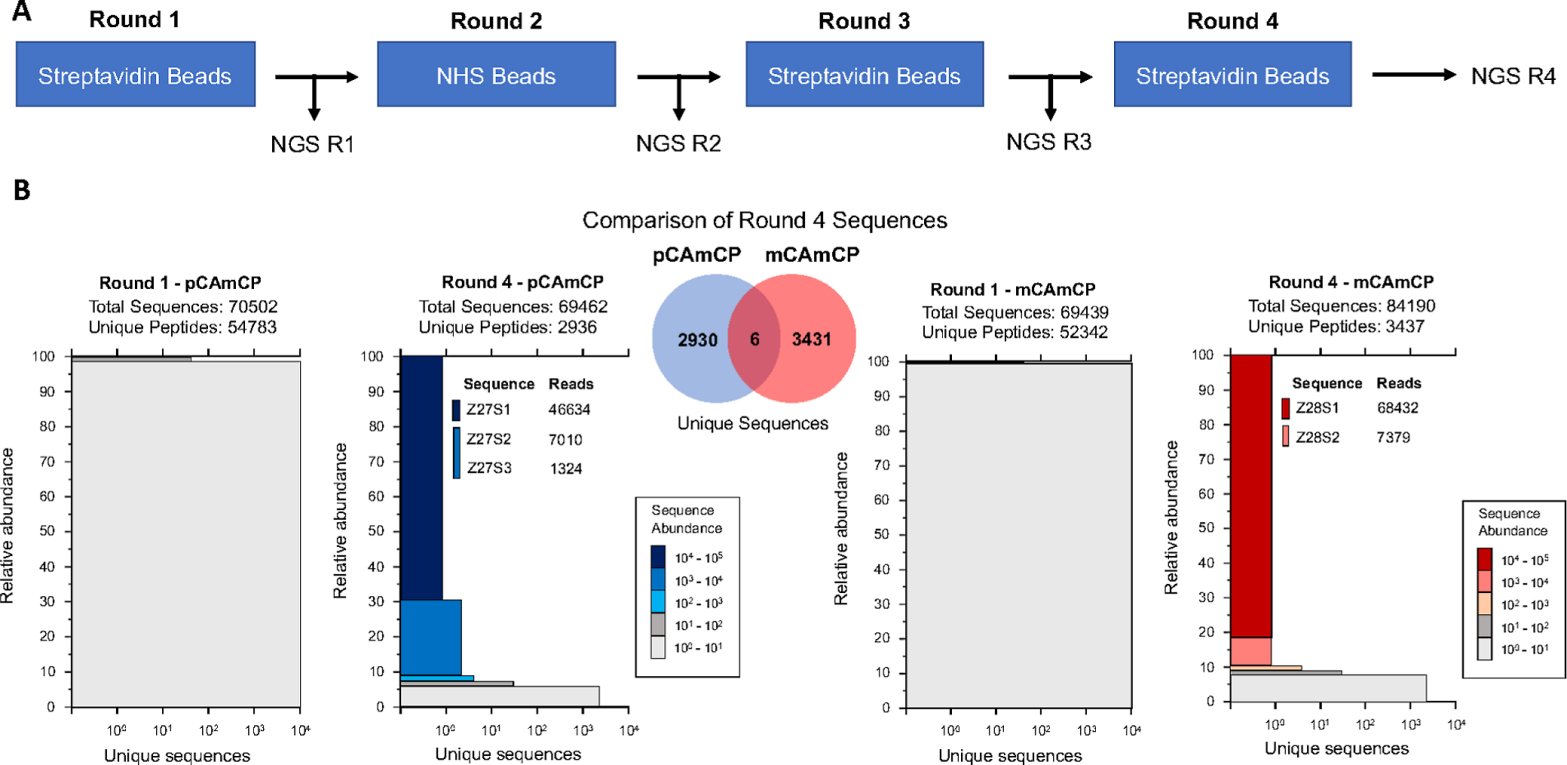
Selection of macrocyclic peptides for binding to ZNRF3. (A) Phages were selected to bind to the ZNRF3 extracellular domain through four rounds of selection with alternating streptavidin and NHS beads to capture the protein target. (B) Illumina next-generation sequencing demonstrated strong enrichment of unique sequences using both pCAmCP (blue) and mCAmCP (red). Comparison of round 4 sequences for each (Venn diagram) demonstrated that the peptide sequences were dependent upon the specific linker for enrichment, with only 6 overlap sequences being found in each selection.

After validating pCAmCP and mCAmCP for the cyclization of phage-displayed peptides, we aimed to further confirm their applicability by using the linkers to identify macrocyclic peptides for a target protein. In addition to simply ensuring the cyclization potential on phage, we also looked to investigate the selection differences using two different linkers to macrocyclize the same initial peptide library. ZNRF3 is a membrane protein that contains an intracellular E3 ligase domain and has previously shown potential in promoting degradation of oncogenic membrane proteins, such as PDL1.^32^ ZNRF3 also plays a major role in the regulation of the Wnt signaling cascade, which can modulate a variety of cell processes related to oncogenesis and cell differentiation.^33−36^ Ligands that bind to ZNRF3, such as R-spondins, have also been shown to promote internalization, making them possibly useful as drug delivery scaffolds.^37^ Because it has an extracellular domain that can be easily accessible to macrocyclic peptides, we envisioned that a selection against the extracellular domain for ZNRF3 (ZNRF3-ECD) would afford peptides that could potentially be used in the development of proteolysis-targeting chimeras (PROTACs) for a variety of membrane proteins. We expressed and characterized a biotinylated ZNRF3-ECD using an Avi-Tag on the N-terminus to facilitate the selection using streptavidin magnetic beads, as has been previously described (Figure S20).^38^ A 12-mer phage-displayed peptide library (CX_12_C) fused to the N-terminus of pIII was expressed and cyclized with either pCAmCP or mCAmCP, then subsequently used for selection against ZNRF3-ECD. To avoid the potential for enriching peptides that bind to the streptavidin resin, we alternated loading the protein using streptavidin and NHS-functionalized magnetic beads for the first three rounds of selection (Figure 5).

To identify peptides that were enriched for binding ZNRF3-ECD, phage genomes from each round of selection were isolated and submitted for Illumina next-generation sequencing. All reads were processed using a previously described paired-end filtering that limits sequencing errors, where any reads with mismatches within the library regions were discarded.^39^ After four rounds of selection, there was strong enrichment of three peptides (Z27S1, Z27S2, and Z27S3) in the pCAmCP selection and two peptides (Z28S1 and Z28S2) in the mCAmCP selection (Figure 5). Interestingly, the enriched peptides were dependent upon the linker used in selection, with less than 0.1% of peptides being shared after round 4 of both selections (Figure 5, Venn diagram). For pCAmCP, the top peptide, Z27S1, composed 67% of the 69,462 total sequences analyzed in round 4 (Figure 5). While it was not found in the round 1 sequencing data, enrichment analysis of Z27S1 after round 2 showed over a 16,000-fold increase in abundance (Figure S21). The lesser enriched peptides Z27S2 and Z27S3 also showed over 8000-fold and 700-fold increases in abundance between rounds 2 and 4, respectively (Figure S21). In the mCAmCP selection, Z28S1 made up 81% of the 84,190 total sequences after round 4, with over 32,000-fold enrichment after round 1 (Figure 5 and Figure S22). One additional peptide, Z28S2, also showed considerable enrichment after round 2 of selection, with an ∼5000-fold increase in abundance (Figure S22).

**Figure 6:**
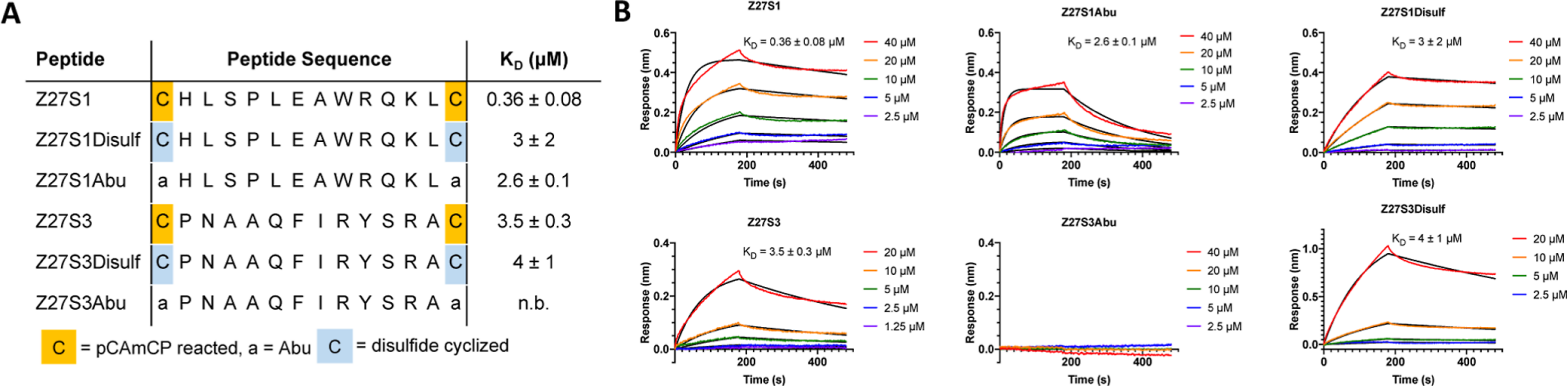
Characterization of peptides for binding to the ZNRF3 extracellular domain. (A) Top peptides from the pCAmCP selection were investigated for their binding to ZNRF3 through biolayer interferometry studies. *K*_D_ values are reported as the mean ± s.d. of three independent experiments (*n* = 3). (B) Representative traces for the biolayer interferometry experiments summarized in panel (A). Raw traces (multicolored by concentration) were fitted (black lines) to a 1:1 protein/ligand binding model to calculate kinetic parameters.

Following identification of peptide sequences through next-generation sequencing, the peptides were synthesized and analyzed for their binding to ZNRF3-ECD through biolayer interferometry assays (see the Supporting Information for synthetic methods, Figures S23–S25 and Tables S1 and S2 for characterization). Biotinylated ZNRF3-ECD was immobilized onto streptavidin biosensors, and then its interaction with each peptide was investigated. Peptide Z27S1 exhibited a high affinity for ZNRF3 with an observed *K*_D_ of 0.36 ± 0.08 μM, while Z27S3 showed a modest affinity (*K*_D_ = 3.5 ± 0.3 μM) (Figure 6A,B). This further validates the observed enrichment data, as Z27S1 gave much higher abundances than Z27S3 during the selection. Unfortunately, Z27S2 was unable to be reliably tested due to poor solubility and aggregation. In the mCAmCP selection, Z28S1 exhibited some binding to ZNRF3, but had extremely quick off rates, resulting in a *K*_D_ > 40 μM, while Z28S2 was also unable to be tested because of poor solubility (Figure S26). Thus, follow-up studies were focused on characterizing Z27S1 and Z27S3 for their affinities for ZNRF3. To better understand the interactions between the selected peptides and ZNRF3, various analogues that resulted in different cyclic or linear scaffolds of Z27S1 and Z27S3 were synthesized (Figure 6A and Figures S27–S30). For Z27S1, the disulfide-cyclized peptide (Z27S1Disulf) exhibited an affinity for ZNRF3 approximately 10-fold lower with a *K*_D_ of 3 ± 2 μM (Figure 6A,B). Additionally, a strictly linear analogue with 2-aminobutyric acid (Abu) in place of the cysteines (Z27S1Abu) also showed a decrease in affinity with a *K*_D_ of 2.6 ± 0.1 μM (Figure 6A,B). Both of these results indicate that Z27S1 is dependent on pCAmCP for binding to ZNRF3. For Z27S3, the disulfide analogue (Z27S3Disulf) also showed modest affinity to the protein with a *K*_D_ of 4 ± 1 μM, which is not significantly different than the pCAmCP-cyclized peptide (Figure 6A,B). However, the Abu derivative, Z27S3Abu, exhibited no binding to ZNRF3 (Figure 6A,B). This indicates that Z27S3 still is required to be cyclic to interact with the protein, but there is less dependence on the linker for its interactions in comparison to Z27S1. To better understand the differential enrichment of peptides depending upon the linker used during selection, we cyclized Z27S1 with mCAmCP (Z27S1-mCP, see Figure S31 for characterization) and tested its affinity with ZNRF3 using biolayer interferometry. In coordination with the next-generation sequencing results that showed Z27S1 at only 0.01% of the total sequences of the library cyclized with mCAmCP after four rounds of selection, Z27S1-mCP exhibited no significant binding to ZNRF3 (Figure S32). This further indicates that despite the minute differences in linker composition, they give altered conformations in the peptide libraries during selection. While future crystallographic and structure–activity relationship studies will provide more detailed analysis of the interactions between the peptides with ZNRF3, these preliminary characterization studies demonstrate the potential of using the CAmCP linkers in phage selections while also highlighting that variations in linker composition can lead to different peptide scaffolds and different enrichment patterns.

## Discussion

We have identified 2-cyanopyrimidines that are functionalized with the 2-chloroacetamide moiety as effective linkers for the macrocyclization of peptides displayed on proteins and the generation of phage-displayed macrocyclic peptide libraries. Two different compounds, pCAmCP and mCAmCP, demonstrated efficient macrocyclization of peptides on the phage without hindering infection. In comparison to the previously developed linkers that react with N-terminal cysteines, the CAmCP compounds contain a more hydrophilic core that could enhance phage display selections under physiological conditions.^12−14^ Also, the CAmCP compounds have the advantage of a simplified synthetic route and a more constrained cyclic scaffold. In previous reports of linkers for bicyclic peptides, the use of hydrophilic linkers facilitated binding of the peptides by allowing for the linker to face away from the protein surface.^15,16^ We envision that these CAmCP derivatives can also give similar benefits to selections, especially when targeting large protein surfaces. This potential for use in phage selections was confirmed by identifying Z27S1, a peptide with a nanomolar affinity to ZNRF3. Also, the selection results demonstrate the importance of having different linkers available for selection of macrocyclic peptides, as the peptide enrichment was dependent on either using pCAmCP or mCAmCP. This is likely due to the cycle size being different between the two libraries. For mCAmCP, the libraries are more constrained due to the chloroacetamide being meta to the nitrile, whereas the chloroacetamide is para in the pCAmCP libraries, giving slightly higher flexibility. This data is also consistent with other reports that highlight the necessity to screen different cycle sizes and linkers to identify potent peptides in phage selections.^10,16,27^ Collectively, the development of these novel linkers will provide researchers with access to even more diverse scaffolds for phage selections of macrocyclic peptides while also improving the hydrophilic properties of currently available linkers.

## Methods

### Primer List

1 pADL-10b-CA5C-for: 5′GCTTCCATGGCCTGCGCAGCAGCAGCAGCATGCGCGGCGAAAGCGGCC3′
2 pADL-10b-NcoI-Rev: 5′GCTTCCATGGCCGGCTGGGCCGCGAGTAAT3′
3 NGS-F1: 5′TCGTCGGCAGCGTCAGATGTGTATAAGAGACAGGCCCAGCCGGCCATG3′
4 NGS-R1: 5′GTCTCGTGGGCTCGGAGATGTGTATAAGAGACAGCGGCCGCTTTCGCCGC3′
5 NGS-i7: 5′CAAGCAGAAGACGGCATACGAGAT[i7]GTCTCGTGGGCTCGG3′
6 NGS-i5: 5′AATGATACGGCGACCACCGAGATCTACAC[i5]TCGTCGGCAGCGTC3′

### Plasmid Construction

#### pBAD-TEV-CA_5_C-sfGFP-His_6_

The plasmid *pBAD-TEV-CA*_*5*_*C-sfGFP-His*_*6*_ was previously constructed and directly used for the expression of protein.^13^

#### pADL-CA_5_C-gIII

Primers pADL-10b-CA5C-for and pADL-10b-NcoI-Rev were used to amplify the template *pADL-AAKAA* using a two-step PCR (8 cycles with annealing at 64 °C, followed by 25 cycles annealing at 72 °C). The PCR products were treated with DpnI at 37 °C for 1 h, then gel-extracted using a 1% agarose gel and a commercially available purification kit (GenCatch). The purified product was then digested with NcoI (37 °C, 1 h, NEBuffer 3.1) and extracted using a PCR purification kit (GenCatch). The digested product was then ligated with T4 Ligase (NEB) at 16 °C for 16 h before being transformed into Top10 Chemical Competent cells (Thermo Fisher), and clones were verified with Sanger sequencing.

#### pADL-(NNK)_12_-gIII

The plasmid *pADL-(NNK)*_*12*_*-gIII* was previously constructed and transformed into electrocompetent *Escherichia coli* ER2738 yielding 1.8 × 10^9^ transformants.^13^

### Expression of CA_5_C-sfGFP

*E. coli* (Top10) containing the plasmid pBAD-TEV-CA_5_C-sfGFP-His_6_ was grown at 37 °C in 1 L of 2× YT containing 100 μg/mL ampicillin to OD_600_ = 0.6. Protein expression was induced by the addition of 0.2% arabinose at 37 °C for 18 h. Cell pellets were then harvested by centrifugation (3750*g*, 20 min), and cells were resuspended in 40 mL of resuspension buffer (50 mM NaH_2_PO_4_, 300 mM NaCl, and 10 mM imidazole, pH 8.0) and lysed by sonication. The lysate was clarified (10,000*g*, 30 min), and the supernatant was incubated with 2 mL of Ni-NTA resin for 1 h at 4 °C. The resin was then washed with 20 mL of resuspension buffer before eluting the protein with elution buffer (resuspension buffer + 250 mM imidazole). The eluent was concentrated and buffer exchanged to TEV protease buffer (50 mM Tris, 150 mM NaCl, and 0.5 mM EDTA, pH 8.0) by centrifugation (MWCO 10 kDa). The protein was then incubated with TEV protease (1:50 enzyme/substrate ratio) for 48 h at 4 °C. Complete digestion was confirmed through mass spectrometry. Following digestion with protease, the protein was concentrated to 1.4 mg mL^–1^ and stored in a storage buffer (TEV protease buffer + 20% glycerol) at −80 °C until further use. Protein concentration was determined by measuring the absorbance of the GFP chromophore at 485 nm using the reported extinction coefficient of 83,300 M^–1^·cm^–1^. The protein characterization and high-resolution mass spectrometry have been previously described.^13^

### Cyclization of CA_5_C-sfGFP with Linkers

Purified CA_5_C-sfGFP was diluted to 500 nM in PBS (pH 7.4) containing 1 mM TCEP and left to incubate for 30 min at 37 °C. Linker (100 μM final concentration) or equivalent amounts of DMSO were then added to the solution, and it reacted at varying times (3 or 16 h) and temperatures (22 or 37 °C). All initial tests for reactivity were performed at 22 °C for 3 h, while linkers pCAmCP and mCAmCP were optimized with the additional conditions. The reaction mixtures were then concentrated and exchanged to ammonium bicarbonate (50 mM, pH 8.0) using an Amicon centrifugal filter (MWCO: 10 kDa). Samples were analyzed using a QExactive Orbitrap LC–MS System (Thermo Fisher) and deconvoluted using UniDec software.^23^

### Peptide Cyclization Kinetics with pCAmCP and mCAmCP

The peptide (CGK-5 or CGK-5Abu, 5 mM stock in water) was diluted to 500 μM in ammonium bicarbonate buffer (50 mM, pH 7.5) containing 1.2 mM TCEP. Following dilution of the peptide, the organic linker (10 mM in DMSO) was added to a final concentration of 550 μM. The reaction mixture was incubated while being shaken at RT. At each time point (5, 10, 15, 30, 45, 60, 120, and 240 min), 5 μL of reaction mixture was removed, and the reaction was quenched by the addition of 5 μL of 100 mM cysteine. Following dilution with 20 μL of water, the sample was then analyzed on a Shimadzu HPLC with an LCMS-2020 mass spectrometer attached (10–40% ACN gradient in H_2_O over 6 min, 0.1% formic acid, 1 mL/min flow rate, Shimadzu C18 250 × 4.6 mm, 5 μm). Peaks corresponding to the cyclized and unreacted forms were then integrated on the Shimadzu LabSolutions software, plotted in GraphPad Prism, and fitted using a one-phase association fitting parameter to determine reaction half-lives.

### Phage Expression and Purification

*E. coli* ER2738 containing the pADL-NNK_12_-gIII or pADL-CA_5_C-gIII phagemid library was grown at 37 °C in 250 mL of 2× YT media supplemented with 100 μg/mL ampicillin, 10 μg/mL tetracycline, and 1% glycerol. Upon reaching OD_600_ = 0.5–0.6, 20 mL of the culture was transferred to a small flask and infected with 20 μL of CM13d3 helper phage (MOI > 5, Antibody Design Laboratories, San Diego, CA) at 37 °C with shaking. After 45 min, the cells were pelleted (3750*g*, 15 min) and resuspended in 200 mL of 2× YT media containing 10 μg/mL tetracycline, 100 μg/mL ampicillin, 25 μg/mL kanamycin, and 1 mM IPTG and incubated at 30 °C. 18 h post-induction, the culture was transferred to 50 mL tubes, cells were pelleted (3750*g*, 20 min), and the supernatant was decanted into new tubes. Phages were precipitated by the addition of appropriate amounts of 5× precipitation buffer (20% polyethylene glycol 8000 and 2.5 M NaCl) to afford a 1× solution and then incubated at 4 °C for 1.5 h. The solution was centrifuged at 10,000*g* for 30 min, then the supernatant was discarded, and the pellet was resuspended in binding buffer (10 mM HEPES, 150 mM NaCl, 10 mM MgCl_2_, and 1 mM KCl, pH 7.4; 2 mL per 50 mL tube). The resuspended phages were combined into one tube, the phage precipitation was repeated, and phages were ultimately resuspended in 2 mL of binding buffer. Any residual bacteria were then pelleted (13,500*g*, 20 min), and the supernatant was transferred to a fresh tube. The solutions were incubated at 65 °C for 15 min to kill any remaining bacteria before being stored at 4 °C until further use.

### Phage Quantification

For all experiments, phages were quantified via a colony-forming unit assay. In this assay, serial dilutions of the phage solution were prepared in 2× YT media, and 10 μL of each dilution was added to 90 μL of log-phase *E. coli* ER2738. Following the addition of the phage dilutions, the culture was incubated at 37 °C for 45 min, and then 10 μL was spotted in triplicate onto agar selection plates containing either 100 μg/mL ampicillin and 10 μg/mL tetracycline, which were incubated at 37 °C overnight. The following day, colonies in each spot were counted, and this number was used to calculate the number of colony-forming units in the solution.

### Phage Infectivity Assay

The purified 12mer phagemid library in binding buffer was incubated with 1 mM TCEP at 37 °C for 30 min. The reduced phages were then reacted with linkers (pCAmCP or mCAmCP) or DMSO equivalents at varying concentrations (50, 100, and 200 μM) and temperatures (22 or 37 °C) for varying times (3 or 24 h). Following incubation under the various conditions, the phage solutions were then titered by using the previously described colony-forming unit assay.

### Biotin Capture Assay

The purified CA_5_C phages (10^11^ to 10^12^ cfu, 98 μL) in binding buffer were incubated with 1 mM TCEP at 37 °C for 30 min. Following incubation, linker (pCAmCP or mCAmCP, 10 mM stock in DMSO, 100 μM final) or equivalent DMSO amounts were added, and the mixture was reacted at RT for 16 h. Phages were precipitated by the addition of 5× PEG/NaCl precipitation buffer (25 μL) and incubated at 4 °C for 1 h before pelleting at 13,500*g* for 15 min. After resuspension of the pellet in 98 μL of binding buffer, Biotin-CBT (10 mM in DMSO, 1% DMSO final) was added to a final concentration of 100 μM. The solution was incubated for 24 h at RT with slow rotation. Following the reaction, the phages were diluted 1000-fold in binding buffer and then were precipitated three times using 5× precipitation buffer before ultimately being resuspended in binding buffer (400 μL). The phages (200 μL) and streptavidin magnetic beads (Cytiva Sera-Mag SpeedBeads, 10 μL/reaction) were then blocked for 30 min using 1% BSA in binding buffer. 100 μL of input phages was removed for titering, and the remaining phages were incubated with the beads for 30 min at RT with shaking before the supernatant was removed and titered. All titers were performed in triplicate. Percent phages captured was calculated by (phages input – phages in supernatant)/phages input × 100.

### Phage Library Cyclization with pCAmCP or mCAmCP

The purified phage library (10^11^ to 10^12^ cfu) in binding buffer was incubated with 1 mM TCEP at 37 °C. After 30 min, the linker (pCAmCP or mCAmCP, 10 mM in DMSO) was added to a final concentration of 100 μM. The solution was incubated for 24 h at RT with slow rotation. The library was then directly used for affinity selection without further purification.

### Expression of ZNRF3

The ZNRF3 ECD was expressed and characterized exactly as previously described.^38^ Plasmid *pET28a-SUMO-ZNRF3-Avi* was cotransformed into *E. coli* (BL21) along with a plasmid expressing BirA. The cells were then inoculated into 2× YT containing 50 μg/mL kanamycin and 34 μg/mL chloramphenicol and grown at 37 °C until OD = 0.5. Expression was then induced by the addition of 1 mM IPTG and 50 μM biotin, and then the protein was expressed for 18 h at 16 °C. The cells were then pelleted (3750*g*, 15 min) and then resuspended in buffer A (50 mM Tris-HCl, 10% glycerol, 150 mM NaCl, and 25 mM imidazole, pH 7.5, 20 mL/liter of expression). The cells were then lysed by sonication (1 s on, 1 s off, 5 min, 60% A, repeated once), and the lysate was clarified by centrifugation (25,000*g*, 20 min). The supernatant was then incubated with a Ni-NTA column for 45 min at 4 °C. The column was then washed with 5 CV of buffer A before being eluted using 2 CV of buffer B (buffer A + 240 mM imidazole). The elution was then concentrated using an Amicon centrifugal filter before being desalted into buffer A on a HiPrep 26/10 column. The SUMO tag was then cleaved by the addition of 30 μg/mL SUMO protease and incubation for 48 h at 4 °C. The protein was then loaded onto a second Ni-NTA column for 45 min at 4 °C, and the flowthrough was collected and concentrated to 1 mL by centrifugation. Pure protein was then isolated using size exclusion chromatography (HiLoad 16/600, Superdex 75 pg) in buffer C (50 mM Tris-HCl, pH 7.5, 10% (v/v) glycerol, 150 mM NaCl, and 0.5 mM EDTA). Pure protein was stored in storage buffer (50 mM Tris-HCl, pH 7.5, 50% (v/v) glycerol, and 150 mM NaCl) at −80 °C before being used for assays.

### Affinity Selection against ZNRF3

#### Phage Selection (Rounds 1, 3, and 4)

Streptavidin-coated magnetic beads (100 μL, 50% slurry, Cytiva Sera-Mag) were transferred to a 1.5 mL tube, washed three times with 1 mL of binding buffer (10 mM HEPES, 150 mM NaCl, 10 mM MgCl_2_, and 1 mM KCl, pH 7.4), resuspended in 100 μL of binding buffer, and split into two tubes. Biotinylated ZNRF3 (12 μg in storage buffer) was added to 500 μL of binding buffer in one of the tubes, and an equal volume of binding buffer was added to the other tube (hereafter referred to as +tube and −tube, respectively). The beads/peptide mixture was incubated with rocking for 15 min at RT. The supernatant was then removed, and the beads were washed with binding buffer (3× 1 mL). 0.25 mL portion of 5× blocking buffer (binding buffer + 5% BSA + 0.5% Tween 20) was added to each tube and phage solution, and they were incubated at RT with end-over-end rotation for 30 min. The blocking buffer was removed from the tube, and the purified phage library was incubated with the beads for 30 min at RT as a negative selection. The supernatant was then transferred to the resin of the +tube and left to incubate at RT for 30 min. After 30 min, the supernatant was removed, and the resin was washed (5× 1 mL) with wash buffer (binding buffer + 0.1% Tween 20) to remove nonspecifically bound phages. During each washing step, the resin was completely resuspended by inversion. To remove phages binding to the polypropylene tube, the resin was transferred to fresh tubes after every other wash. After the last wash, phages were eluted by incubating them for 15 min with 100 μL of elution buffer (50 mM glycine, pH 2.2). The supernatant was then removed and immediately added to 50 μL of neutralization buffer (1 M Tris, pH 8.0). The neutralized elution was immediately used for amplification.

#### Phage Selection (Round 2)

NHS-functionalized magnetic beads (100 μL, Cytiva, NHS Mag Sepharose) were washed with 1 mL of equilibration buffer (1 mM HCl, cold) and then separated into two tubes (±). To the positive tube, 12 μg of ZNRF3 (0.19 mg mL^–1^, 0.2 M NaHCO_3_, and 500 mM NaCl, pH 8.3) was added and incubated for 15 min at RT. The supernatant was then removed, and the residual active groups were blocked by six alternative washes of buffer 1 (0.5 M ethanolamine and 0.5 M NaCl, pH 8.3) and buffer 2 (0.1 M NaAcO and 0.5 M NaCl, pH 4.0). During the second wash with buffer 1, the beads were incubated for 15 min at RT. Following blocking with buffers 1 and 2, the beads and phages were then incubated with 1× blocking buffer (binding buffer + 0.1% Tween 20 + 1% BSA) for 30 min at RT. Phages were added to the negative tube and incubated for 30 min with shaking. The supernatant was removed and then added to the positive tube for a 30 min incubation with shaking. The bound phages were then washed with wash buffer (5× 1 mL) and transferred into a fresh tube after every other wash. Bound phages were then eluted for 15 min in 100 μL of elution buffer (0.1 M glycine, pH 2.2), and the supernatant was neutralized with 50 μL of 1 M Tris, pH 8.0. Eluted phages were then directly used for amplification.

#### Phage Amplification

A small aliquot (10 μL) of the phage elution was removed for the quantification of phages. The remaining solution was added to an actively growing culture of *E. coli* ER2738 (OD_600_ = 0.5–0.6) in 20 mL 2× YT containing 10 μg/mL tetracycline for 45 min at 37 °C with rotation. After 45 min, the cells were pelleted (3750*g*, 15 min), resuspended in 200 mL of 2× YT containing 100 μg/mL ampicillin and 10 μg/mL tetracycline, and amplified overnight at 37 °C. The following day, phagemids were extracted from the amplified culture using a commercial plasmid purification kit. The remaining culture was then inoculated into 100 mL of fresh 2× YT containing 100 μg/mL ampicillin and 10 μg/mL tetracycline for subsequent phage expression.

### Illumina Sequencing of Selected Phage Libraries

A four-step PCR cycle was used to amplify the library region out of the original phagemid library using primers NGS-F1 and NGS-R1, as has been previously described.^39^ The amplicons were purified and extracted from a 3% agarose gel according to a GenCatch gel extraction kit, and then indices were attached using a subsequent PCR with NGS-i7 and NGS-i5 primers. To identify the rounds of selection, each round contained a unique combination of i7 or i5 indices. The PCR products were purified using a GenCatch gel extraction kit and submitted to the Genomics and Bioinformatics center at Texas A&M University for sequencing on an Illumina iSeq (4 M Reads, 2× 150 bp). Sequences underwent paired-end filtering and were analyzed for enrichment in R. All scripts are available in the Supporting Information.

### Biolayer Interferometry Assays

Biolayer interferometry (BLI) experiments were performed using streptavidin-coated biosensors (Sartorius Bio) on an Octet R8 biolayer interferometer (Sartorius Bio). All experiments were performed in assay buffer (10 mM HEPES, 150 mM NaCl, and 0.01% Tween 20, pH 7.5). Biotinylated ZNRF3 (28 μg/mL in assay buffer) was loaded onto the sensor to 4 nm of loading over 10 min, and then the sensors were quenched for one min with a 1% BSA solution in assay buffer. Peptides were serially diluted in assay buffer containing 0.8% DMF, and the association and dissociation to ZNRF3 were measured over 3 and 5 min, respectively. Between each experiment, sensors were regenerated using 10 mM glycine, pH 1.5. Binding curves were double-referenced against a protein-only sensor and a parallel unloaded sensor to correct for any nonspecific binding to the sensors. All curves were fit in the Octet Kinetic Analysis program (Sartorius Bio) using a 1:1 protein/ligand binding model.

## Data Availability

Illumina sequencing data have been deposited into the NCBI database as BioProject PRJNA1144996. All other data are readily available within the main text or Supporting Information.

## Supporting Information Available

Synthetic methods, peptide synthesis, NMR and HRMS data, LC-MS data, and R scripts (PDF)

## Notes

Code Availability Statement: The R scripts for analysis of next-generation sequencing data are available in the Supporting Information.
