# Supplementary material for: 2-Cyanopyrimidine-Containing Molecules for N-Terminal Selective Cyclization of Phage-Displayed Peptides
Source: ACS Chem Biol. 2025 Jan 7;20(1):219–28. doi: 10.1021/acschembio.4c00725 (PMC11744668; doi:10.1021/acschembio.4c00725)
Supplement: Supplementary file 1 — cb4c00725_si_001.pdf [file cb4c00725_si_001.pdf]

SUPPORTING INFORMATION

2-Cyanopyrimidine-Containing Molecules for *N*-Terminal Selective Cyclization of Phage-Displayed Peptides

J. Trae Hampton[1,*], Connor R. Dobie[1], Demonta D. Coleman[1], Moulay I. Cherif[1], Sukant Das[1], and Wenshe Ray Liu[1,2,3,4,*]

[1]Texas A&M Drug Discovery Center, Department of Chemistry, Texas A&M University, College Station, TX 77843, USA

[2]Institute of Biosciences and Technology and Department of Translational Medical Sciences, College of Medicine, Texas A&M University, Houston, TX 77030, USA

[3]Department of Biochemistry and Biophysics, Texas A&M University, College Station, TX 77843, USA

[4]Department of Cell Biology and Genetics, College of Medicine, Texas A&M University, College Station, TX 77843, USA

*Correspondence should be addressed to Wenshe Ray Liu and J. Trae Hampton:

wsliu2007@tamu.edu, jhampton1@tamu.edu

SUPPLEMENTARY METHODS

Synthetic Methods

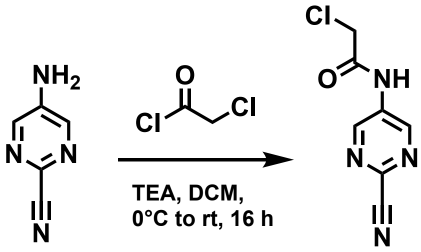

1 pCAmCP

2-chloro-*N*-(2-cyanopyrimidin-5-yl)acetamide (**pCAmCP**): To a stirred solution of 5-aminopyrimidine-2-carbonitrile **1** (100 mg, 0.8 mmol) in DCM (2 ml), triethylamine (0.23 ml, 1.6 mmol) was added and then 2-chloroacetyl chloride (134 mg, 1.2 mmol) was added at 0 °C and left to react at rt for 16 h. The reaction mixture was quenched with water and extracted with DCM (2x20 mL). The combined DCM layers were dried over Na_2SO_4, concentrated and purified by flash chromatography (0-10% MeOH/DCM) providing an off white solid (30 mg, 18%). ^{1}H NMR (400 MHz, DMSO-d_6) δ 11.19 (s, 1H), 9.16 (s, 2H), 4.41 (s, 2H). ^{13}C NMR (101 MHz, DMSO-d_6) δ 167.77, 149.40, 139.24, 137.70, 117.65, 44.67. HRMS (ESI-) $C_7H_4ClN_4O$ 195.0070 [M-H]$^-$

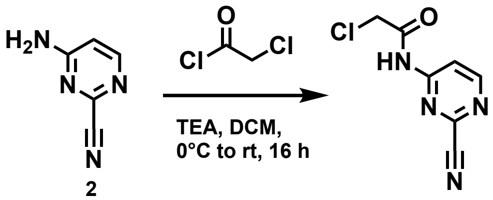

2 mCAmCP

2-chloro-*N*-(2-cyanopyrimidin-4-yl)acetamide (**mCAmCP**): To a stirred solution of 4-aminopyrimidine-2-carbonitrile **2** (100 mg, 0.8 mmol) in DCM (2 ml), triethylamine (0.23 ml, 1.6 mmol) was added and then 2-chloroacetyl chloride (134 mg, 1.2 mmol) was added at 0 °C and left to react at rt for 16 h. The reaction mixture was quenched with water and extracted with DCM (2x20 mL). The combined DCM layers were dried over Na_2SO_4, concentrated and purified by flash chromatography (0-10% MeOH/DCM) followed by prep-TLC provided off white solid (10 mg). ^{1}H NMR (400 MHz, DMSO) δ 11.76 (s, 1H), 8.85 (d, *J* = 5.9 Hz, 1H), 8.26 (d, *J* = 5.9 Hz, 1H),

4.42 (s, 2H). ^{13}C NMR (101 MHz, DMSO) δ 167.53, 160.39, 158.62, 143.87, 116.13, 113.15, 43.89. HRMS (ESI-) $C_7H_4ClN_4O$ 195.0070 $[M-H]^-$

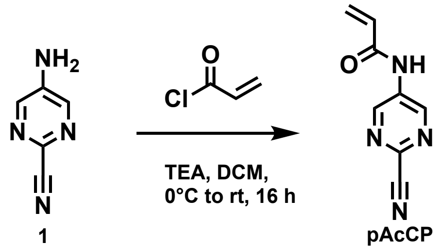

N-(2-cyanopyrimidin-5-yl) acrylamide (**pAcCP**): To a stirred solution of 5-aminopyrimidine-2-carbonitrile **1** (100 mg, 0.8 mmol) in DCM (2 ml), triethylamine (0.23 ml, 1.6 mmol) was added and then acryloyl chloride (108 mg, 1.2 mmol) was added at 0 °C and left to react at rt for 16 h. The reaction mixture was quenched with water and extracted with DCM (2x20 mL). The combined DCM layers were dried over Na_2SO_4, concentrated and purified by flash chromatography (0-10% MeOH/DCM) providing pure compound as an off white solid (60 mg, 41%). ^1H NMR (400 MHz, DMSO-d_6) δ 11.09 (s, 1H), 9.27 (s, 2H), 6.59 – 6.40 (m, 2H), 6.01 (dd, *J* = 9.7, 2.1 Hz, 1H). ^{13}C NMR (101 MHz, DMSO-d_6) δ 164.79, 148.23, 137.17, 130.79, 130.21, 116.69. HRMS (ESI-) $C_8H_5N_4O$ 173.0456 $[M-H]^-$

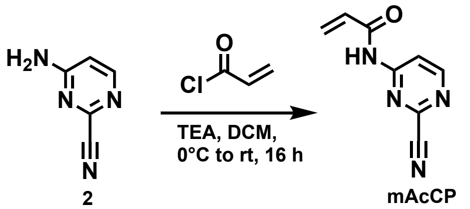

N-(2-cyanopyrimidin-4-yl)acrylamide (**mAcCP**): To a stirred solution of 4-aminopyrimidine-2-carbonitrile **2** (100 mg, 0.8 mmol) in DCM (2 ml), triethylamine (0.23 ml, 1.6 mmol) was adedd and then acryloyl chloride (108 mg, 1.2 mmol) was added at 0 °C and leave to rt for 16 h. The reaction mixture was quenched with water and extracted with DCM (2x20 mL). The combined DCM layers were dried over Na_2SO_4, concentrated and purified by flash chromatography (0-10% MeOH/DCM), followed by prep-TLC provided off white solid (10 mg). ^1H NMR (400 MHz, DMSO-d_6) δ 11.63 (s, 1H), 8.84 (d, *J* = 5.9 Hz, 1H), 8.39 (d, *J* = 5.9 Hz, 1H), 6.59 (dd, *J* = 17.0, 10.1 Hz, 1H), 6.42 (dd, *J* = 17.0, 1.7 Hz, 1H), 5.94 (dd, *J* = 10.1, 1.7 Hz, 1H). ^{13}C NMR (101 MHz, DMSO-d_6) δ 165.41, 160.15, 159.10, 143.88, 130.95, 130.77, 116.18, 113.30. HRMS (ESI-) $C_8H_5N_4O$ 173.0459 $[M-H]^-$

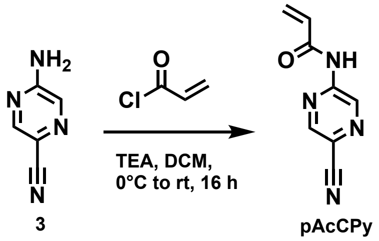

N-(5-cyanopyrazin-2-yl)acrylamide (**pAcCPy**): To a stirred solution of 5-aminopyrazine-2-carbonitrile **1** (100 mg, 0.8 mmol) in DCM (2 ml), triethylamine (0.23 ml, 1.6 mmol) was added and then acryloyl chloride (108 mg, 1.2 mmol) was added at 0 °C and left to react at rt for 16 h. The reaction mixture was quenched with water and extracted with DCM (2x20 mL). The combined DCM layers were dried over Na_2SO_4, concentrated and purified by flash chromatography (0-10% MeOH/DCM) provided off white solid (30 mg). ^{1}H NMR (400 MHz, DMSO-d_6) δ 11.57 (s, 1H), 9.54 (d, J = 1.5 Hz, 1H), 9.00 (d, J = 1.5 Hz, 1H), 6.64 (dd, J = 17.0, 10.2 Hz, 1H), 6.43 (dd, J = 17.0, 1.7 Hz, 1H), 5.94 (dd, J = 10.2, 1.7 Hz, 1H). ^{13}C NMR (101 MHz, DMSO-d_6) δ 164.72, 151.12, 148.14, 137.53, 130.75, 123.73, 116.87. HRMS (ESI-) $C_8H_5N_4O$ 173.0458 [M-H]$^-$

Peptide Synthesis

Automated Peptide Synthesis

Initial sequences of peptides were synthesized on a low loading ProTide rink amide resin (CEM #R002) using a MultiPep 2 Peptide Synthesizer (CEM). All amino acid derivatives were standard derivatives for Fmoc peptide synthesis commercially purchased from Chem-Impex. Fmoc-amino acids were deprotected using 20% piperidine in DMF (2 x 2 mL, 5 min at 50 °C). Amino acids were coupled using HATU/NMM double coupling cycles (4.2 eq amino acid, 4.2 eq HATU, 8 eq NMM, 15 min at 50 °C). Following synthesis, the resin was dried with ethanol washes (3 x 3 mL) followed by dichloromethane washes (3 x 3 mL) and evaporated under vacuum.

Peptide Cleavage

Peptides were cleaved in 2 mL of 92.5:2.5.2.5:2.5 TFA:TIS:DODT:H_2O for 3 hours at room temperature. Following cleavage, the resin was filtered, and peptides were precipitated from the filtrate by an 8-fold dilution in cold diethyl ether. After collection by centrifugation (3750 x*g*,

15 min), they were lyophilized to a powder overnight. They were then dissolved in DMF and purified via preparative LC-MS (see conditions in Table S2).

Cyclization of Peptides with CAmCP Linkers

5 mM stocks of purified linear peptides were prepared in DMF. Peptides were then diluted to 500 µM in reaction buffer (50 mM ammonium bicarbonate, 1.2 mM TCEP, pH 7.2) and reacted with 550 µM of linker (pCAmCP for Z27 peptides, mCAmCP for Z28 peptides) for 24 h with shaking at room temperature. The mixture was then diluted in water and lyophilized prior to purification with HPLC-MS. Refer to conditions in Table S2 for purification conditions of each peptide.

Oxidation of Disulfide-Cyclized Peptides

Linear peptides containing two cysteines were oxidized by dissolving them in 1 mL of 1:2:2 DMSO:ACN:H_2O and incubated for 72 h at room temperature with shaking. The oxidized peptides were then directed purified using a Shimadzu LC-MS 2020 system (refer to Table S2 for conditions).

SUPPLEMENTARY FIGURES

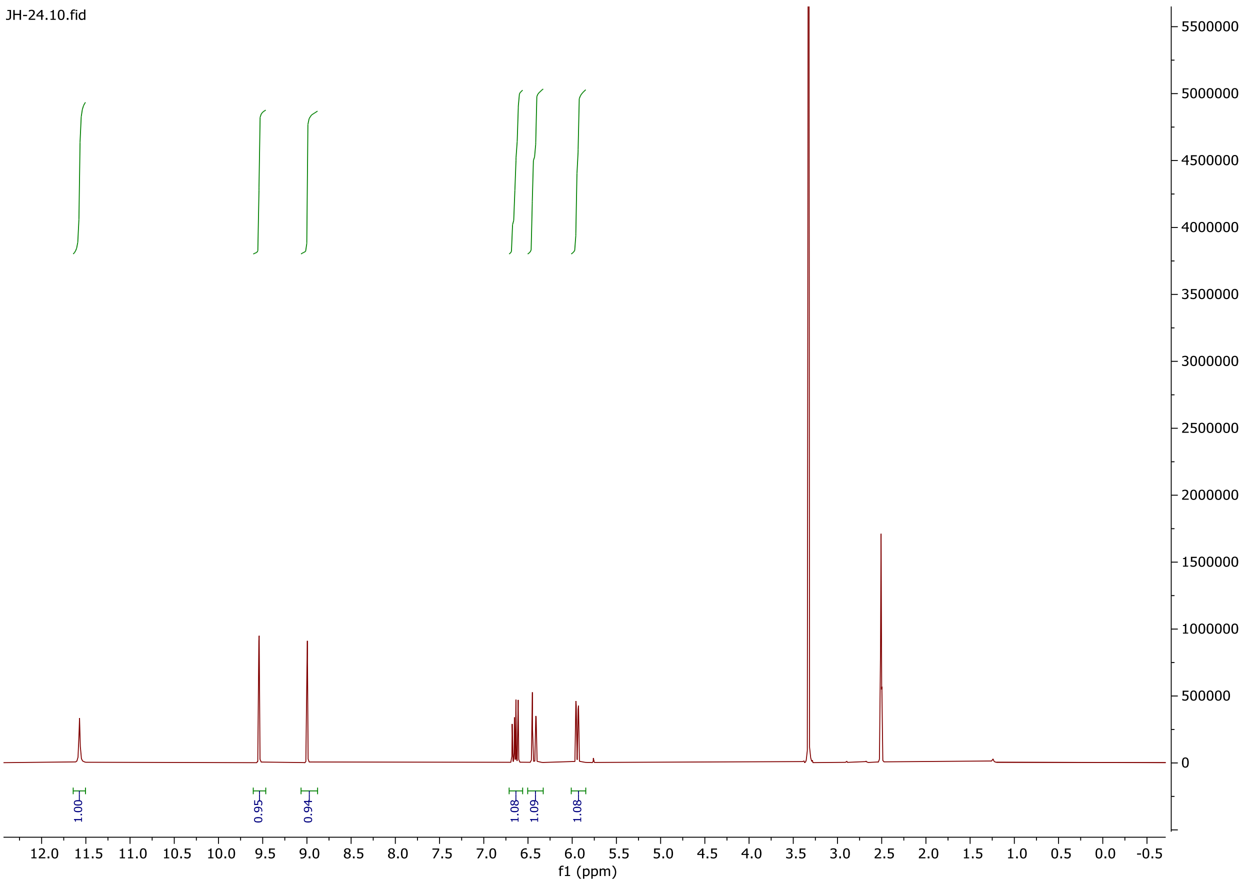

Figure S1: ^{1}H NMR for pAcCPy

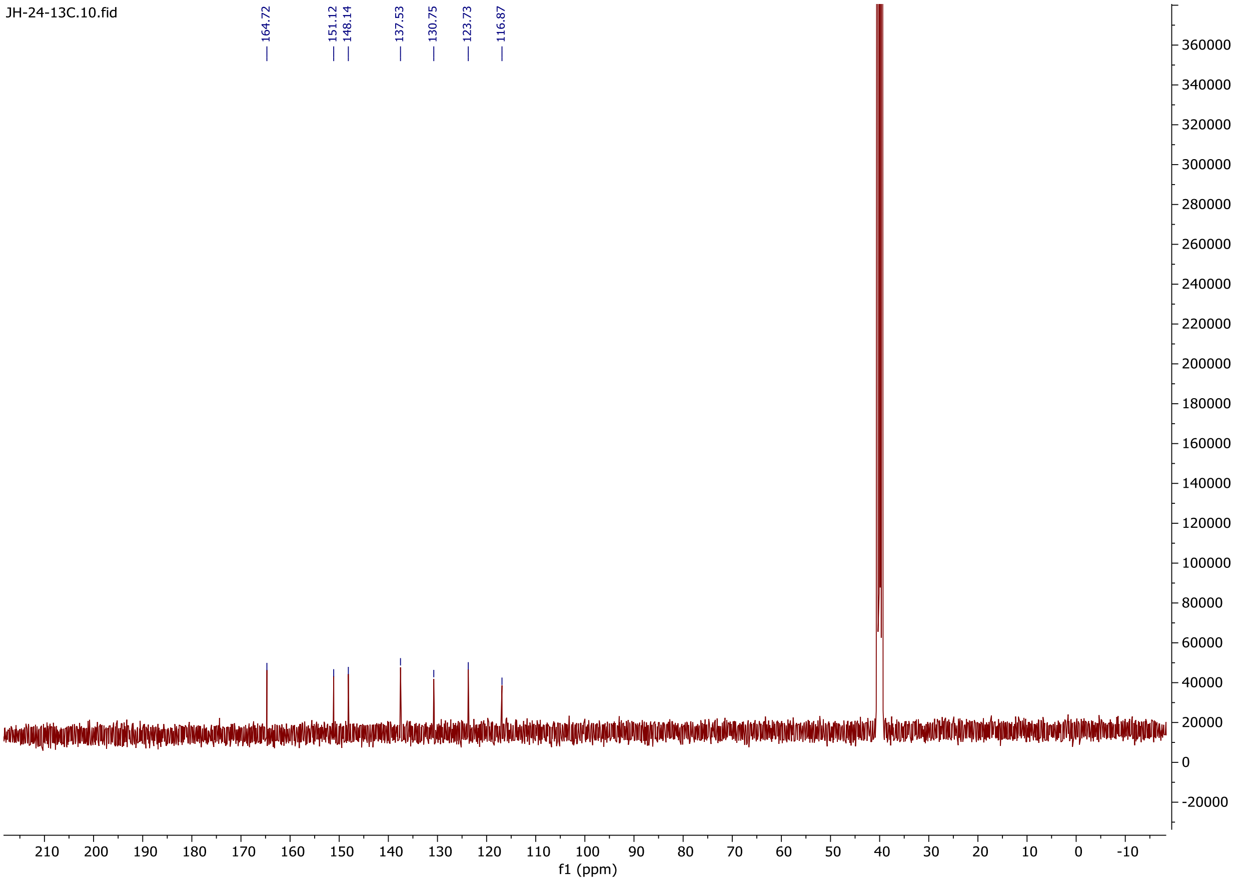

Figure S2: ^{13}C NMR for pAcCPy

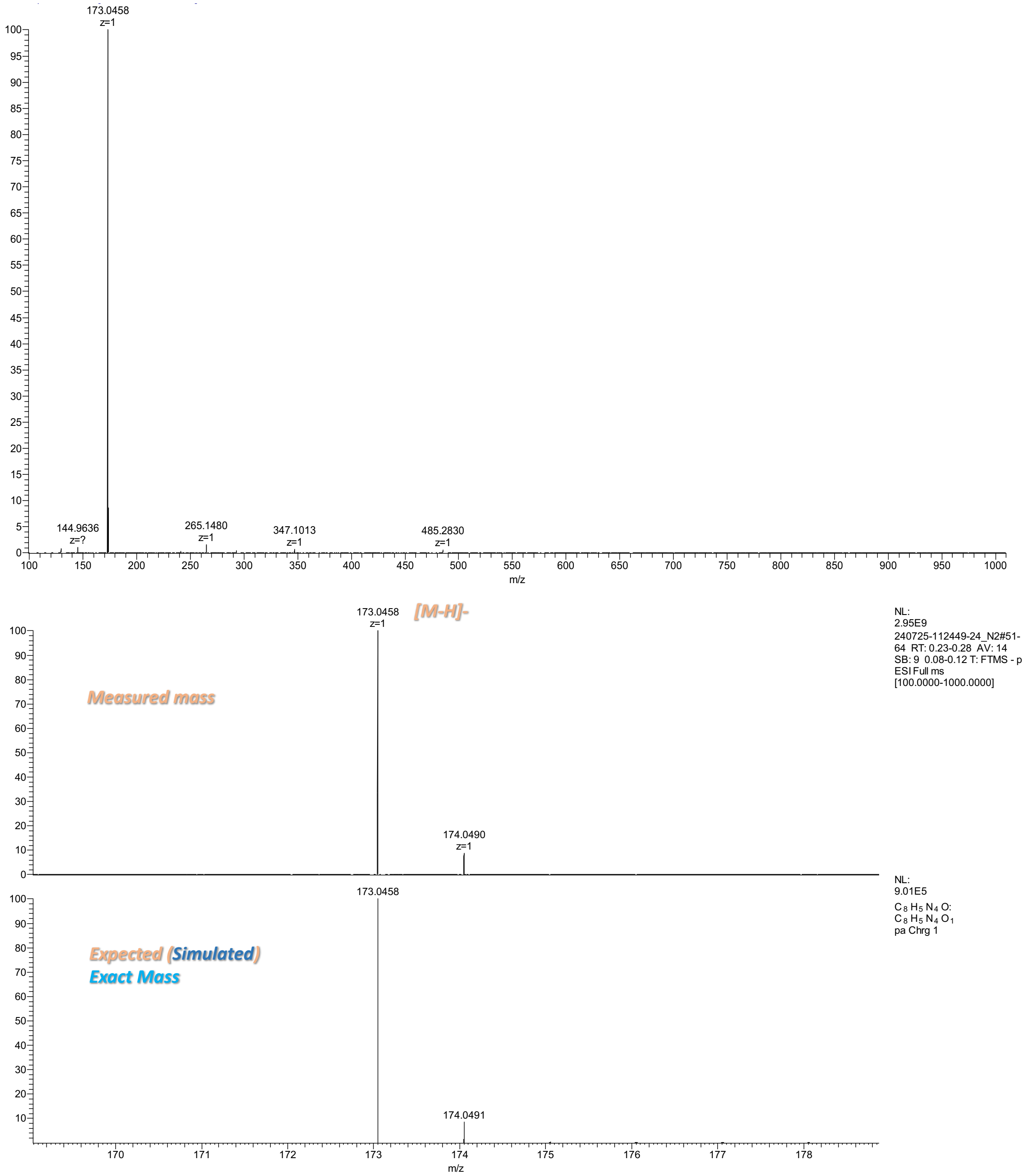

Figure S3: HRMS data for pAcCPy. The top spectra corresponds to the TIC from a direct injection ranging from 100-1000 (m/z) in negative mode (ESI). The bottom gives a comparison between the observed and expected mass isotope distribution.

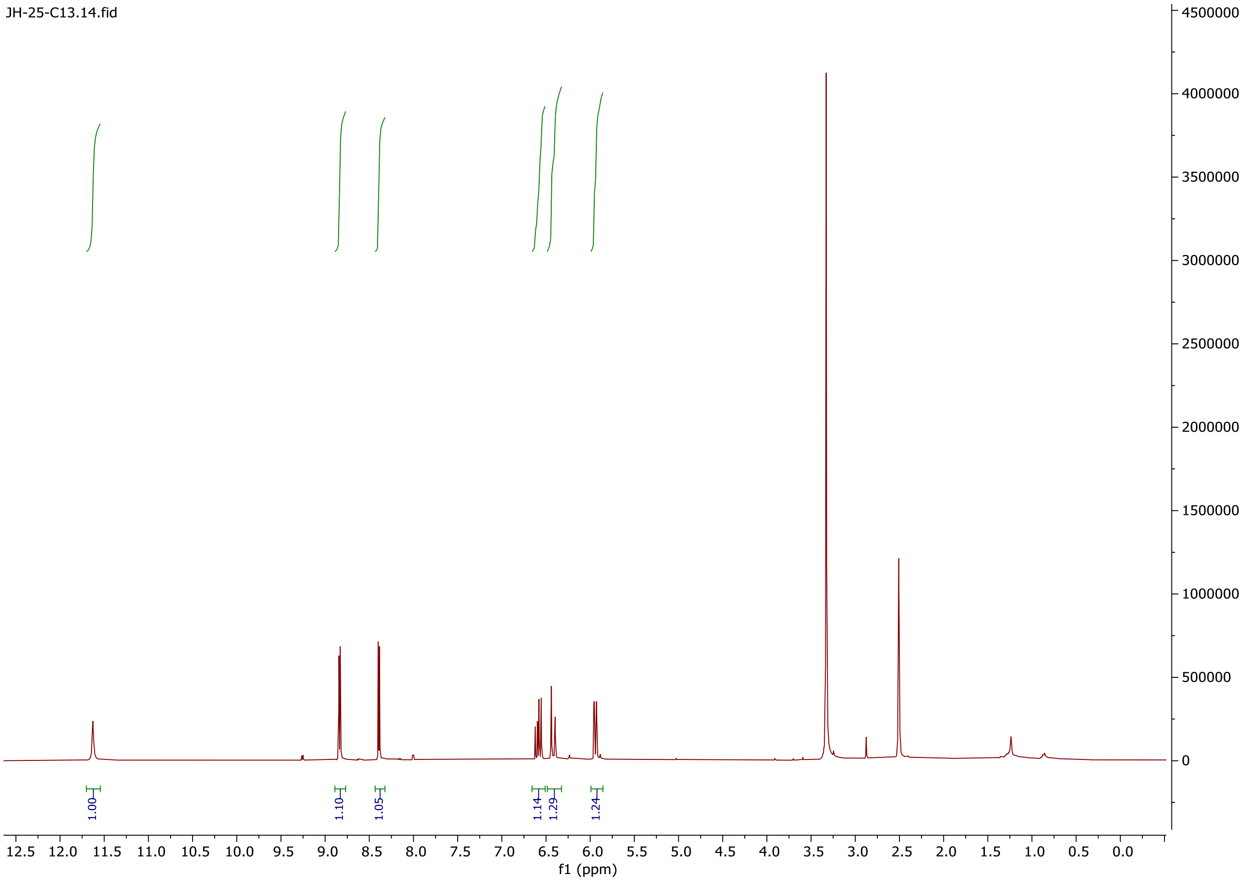

Figure S4: ^{1}H NMR for mAcCP.

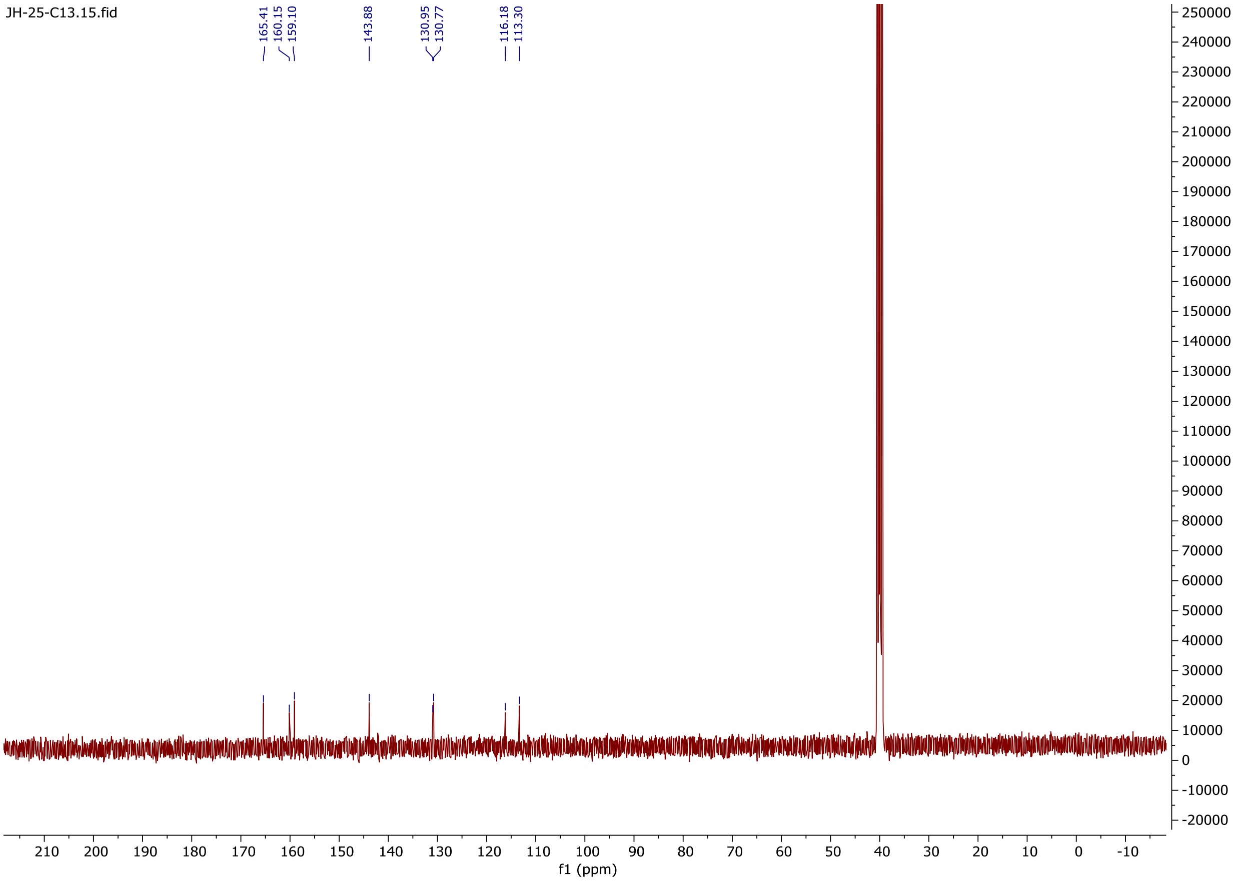

Figure S5: ^{13}C NMR for mAcCP.

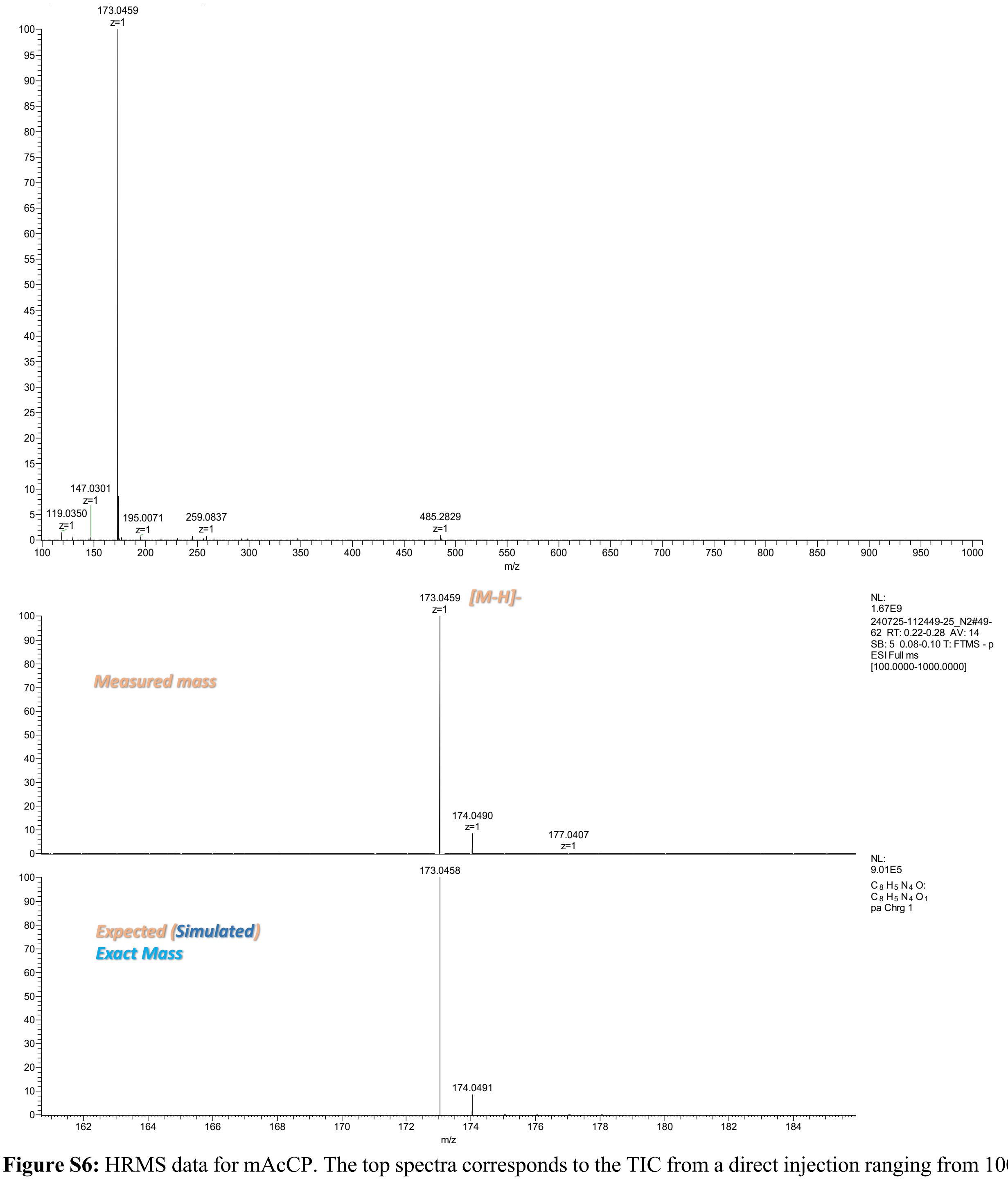

Figure S6: HRMS data for mAcCP. The top spectra corresponds to the TIC from a direct injection ranging from 100-1000 (m/z) in negative mode (ESI). The bottom gives a comparison between the observed and expected mass isotope distribution.

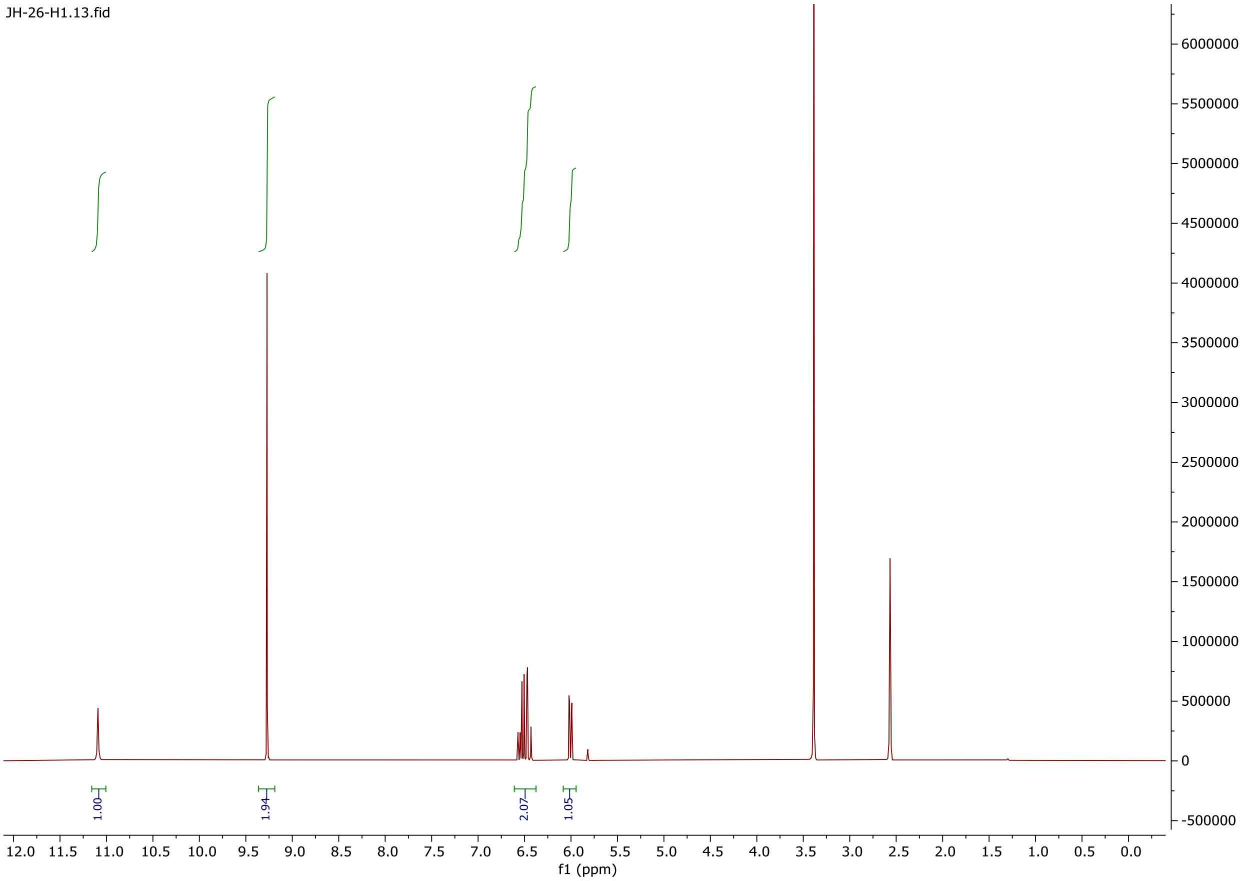

Figure S7: ^{1}H NMR for pAcCP.

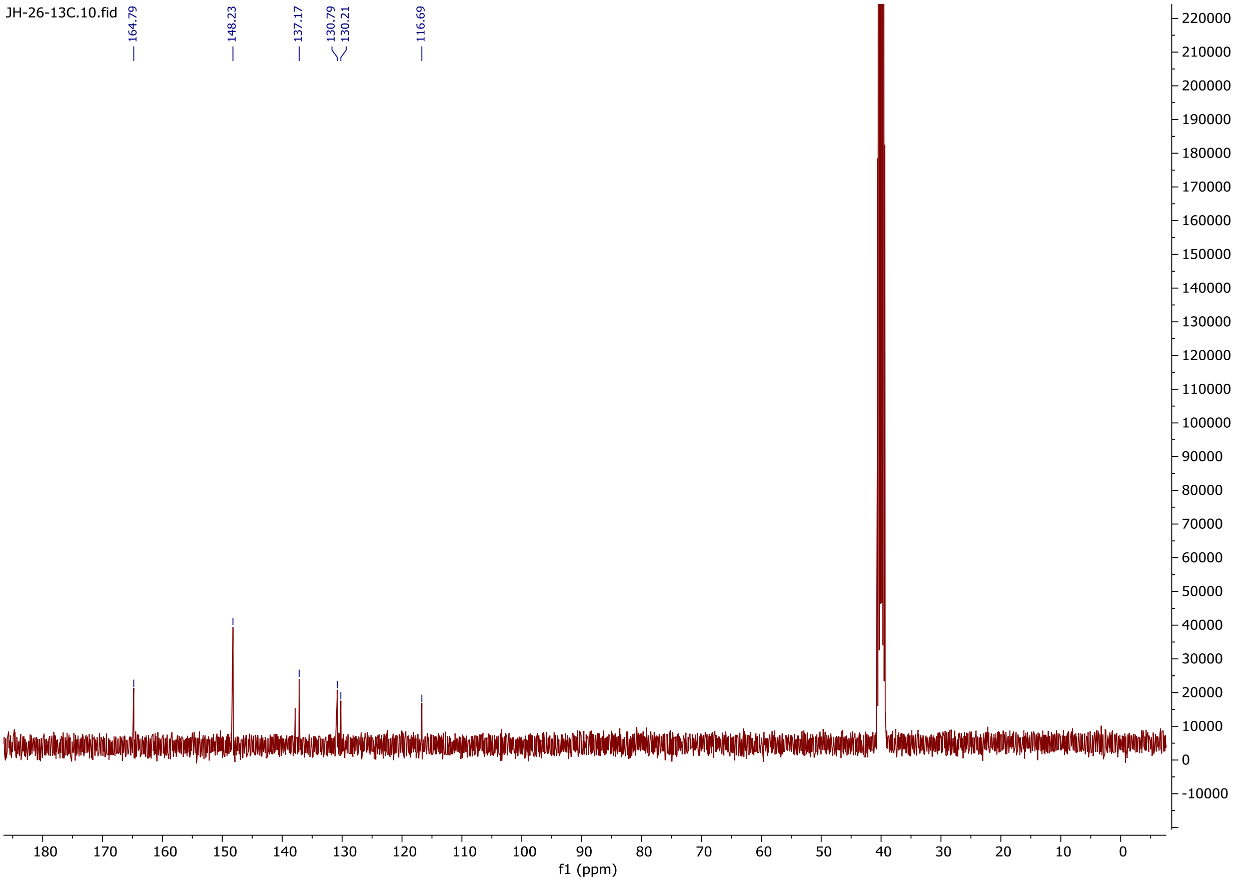

Figure S8: ^{13}C NMR for pAcCP.

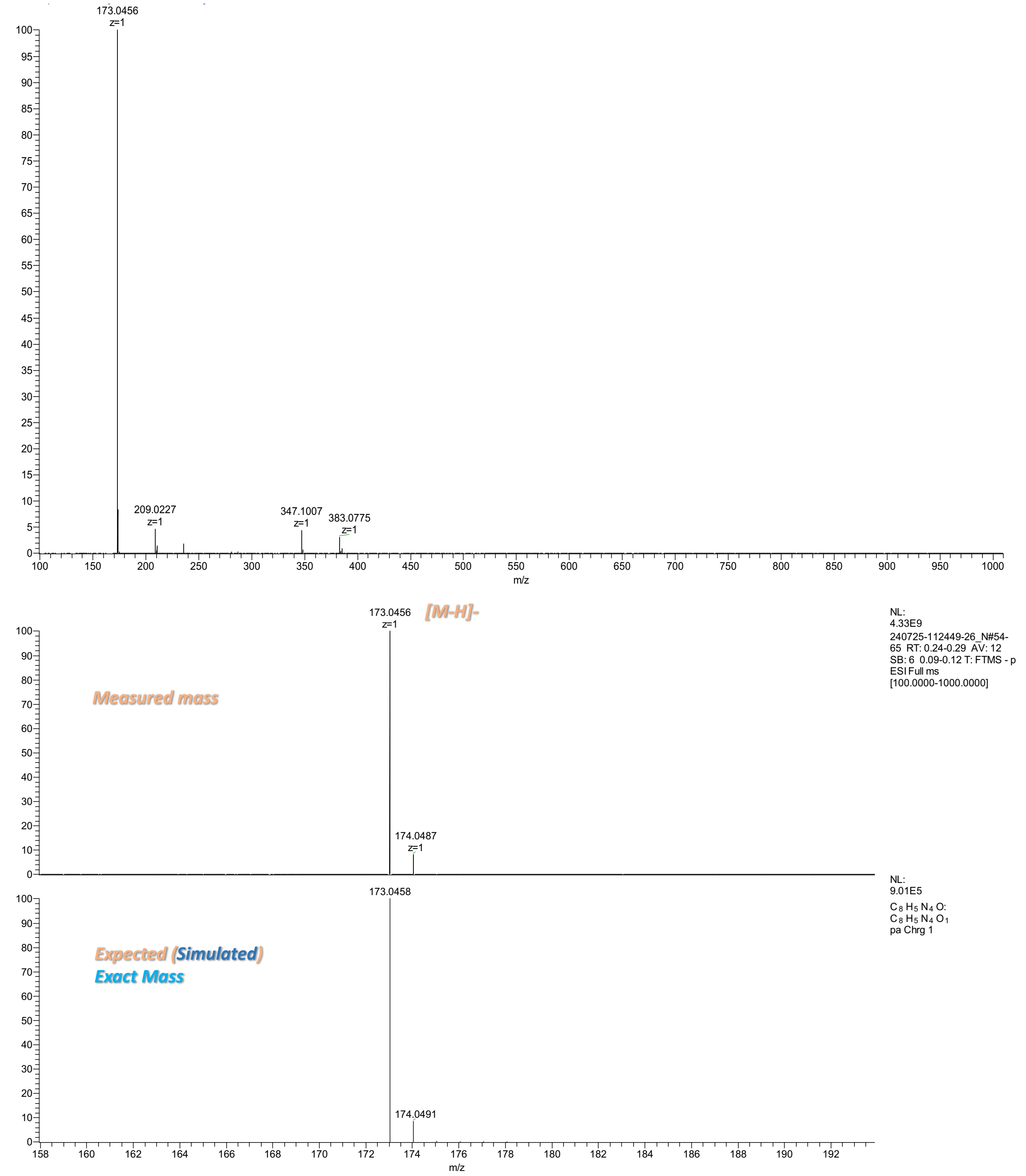

Figure S9: HRMS data for pAcCP. The top spectra corresponds to the TIC from a direct injection ranging from 100-1000 (m/z) in negative mode (ESI). The bottom gives a comparison between the observed and expected mass isotope distribution..

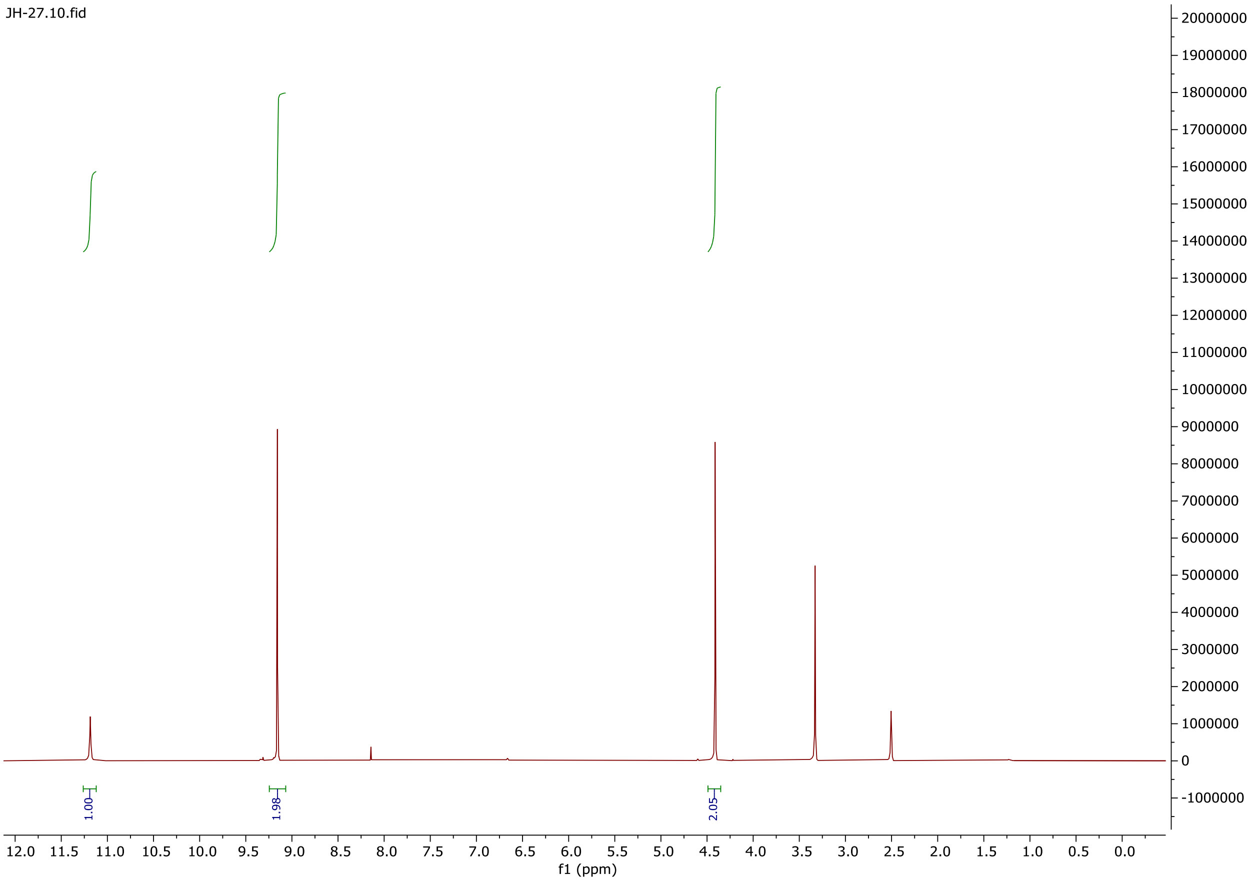

Figure S10: ^{1}H NMR for pCAmCP.

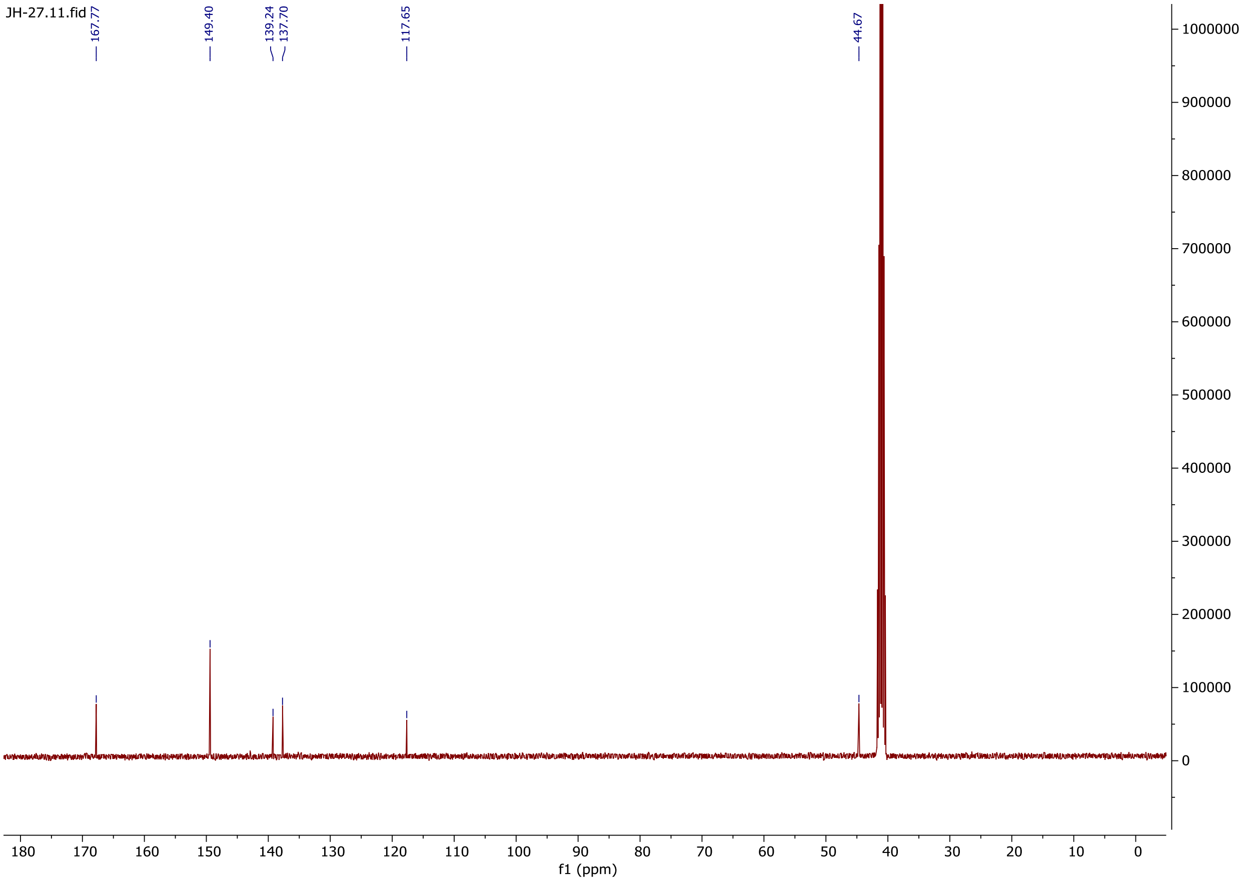

Figure S11: ^{13}C NMR for pCAmCP.

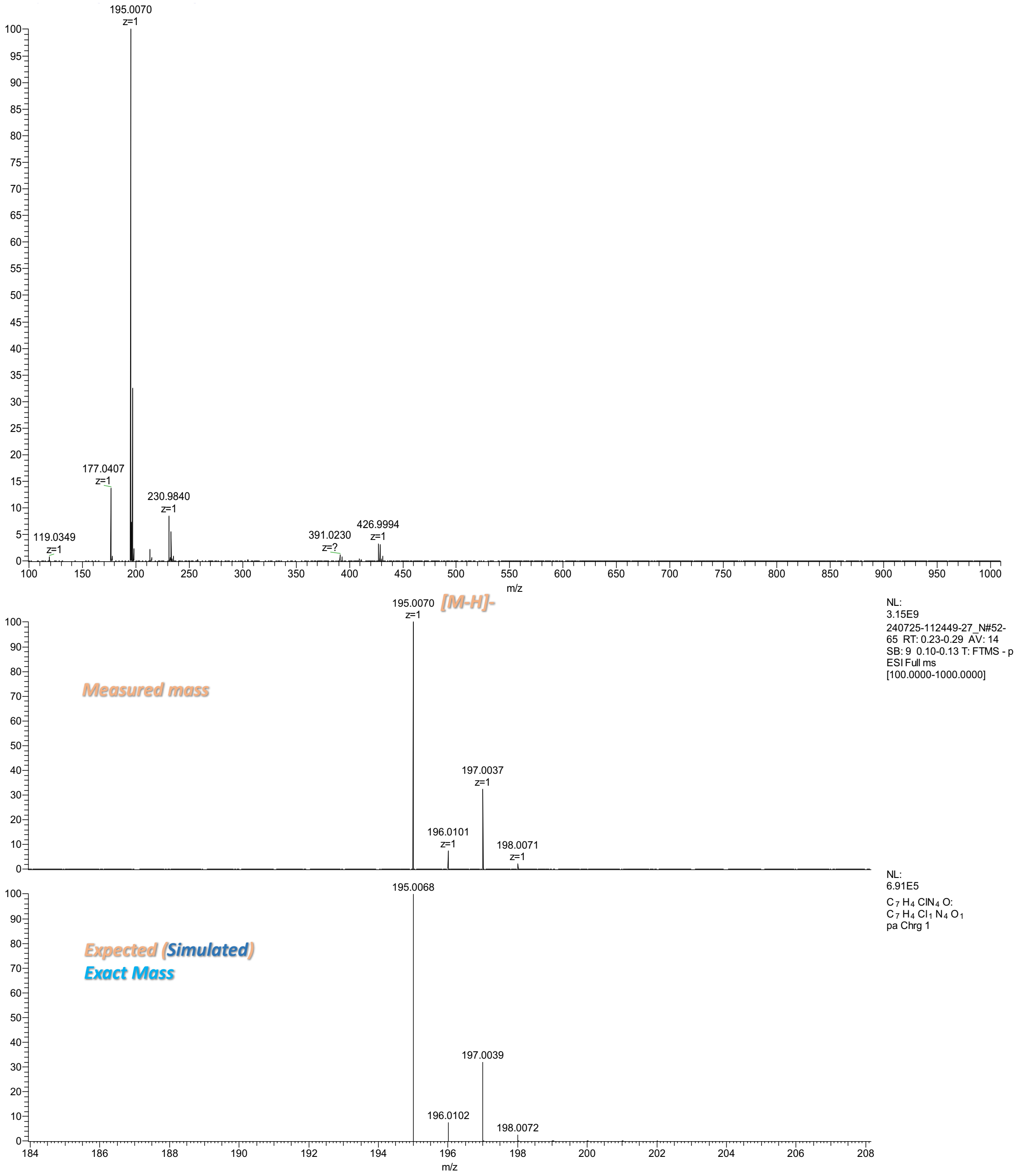

Figure S12: HRMS data for pCAmCP. The top spectra corresponds to the TIC from a direct injection ranging from 100-1000 (m/z) in negative mode (ESI). The bottom gives a comparison between the observed and expected mass isotope distribution.

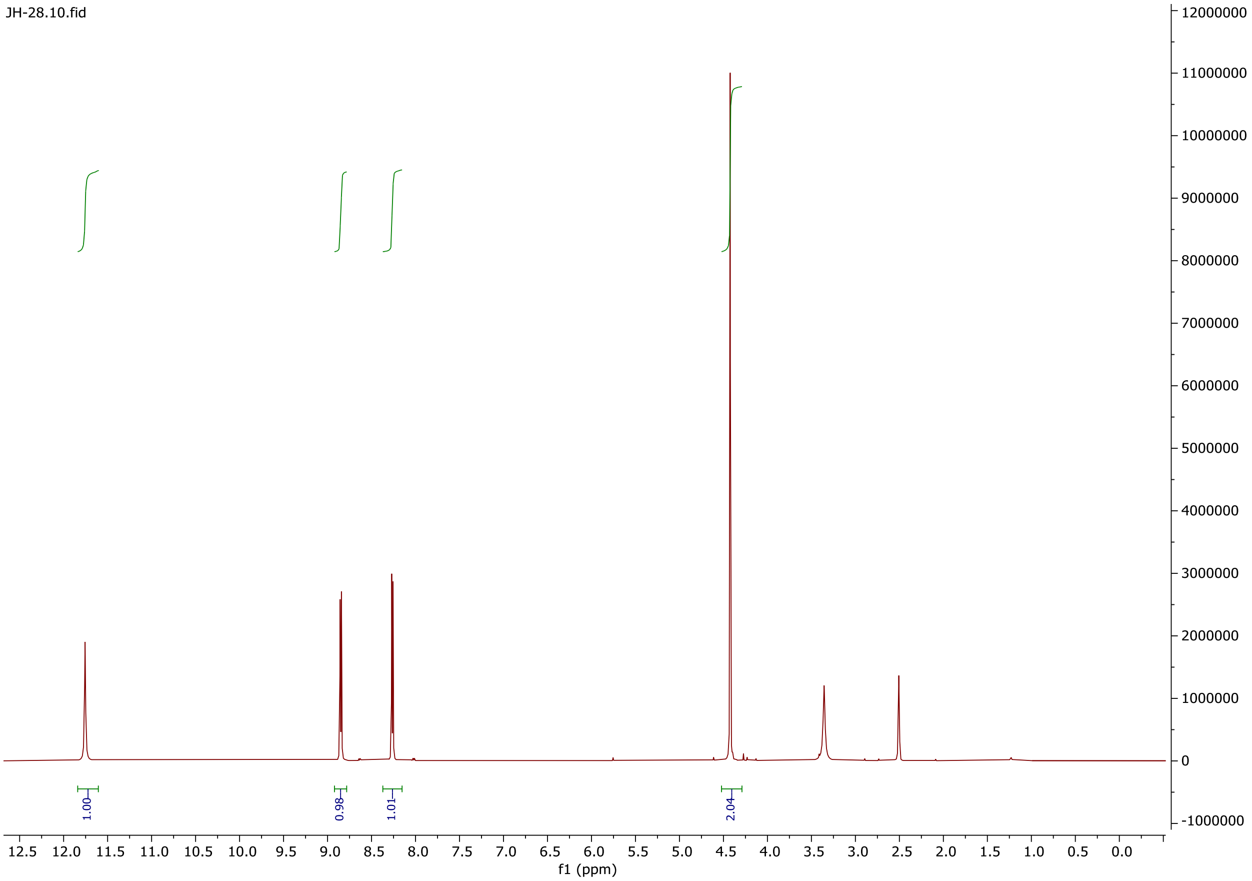

Figure S13: ^{1}H NMR for mCAmCP

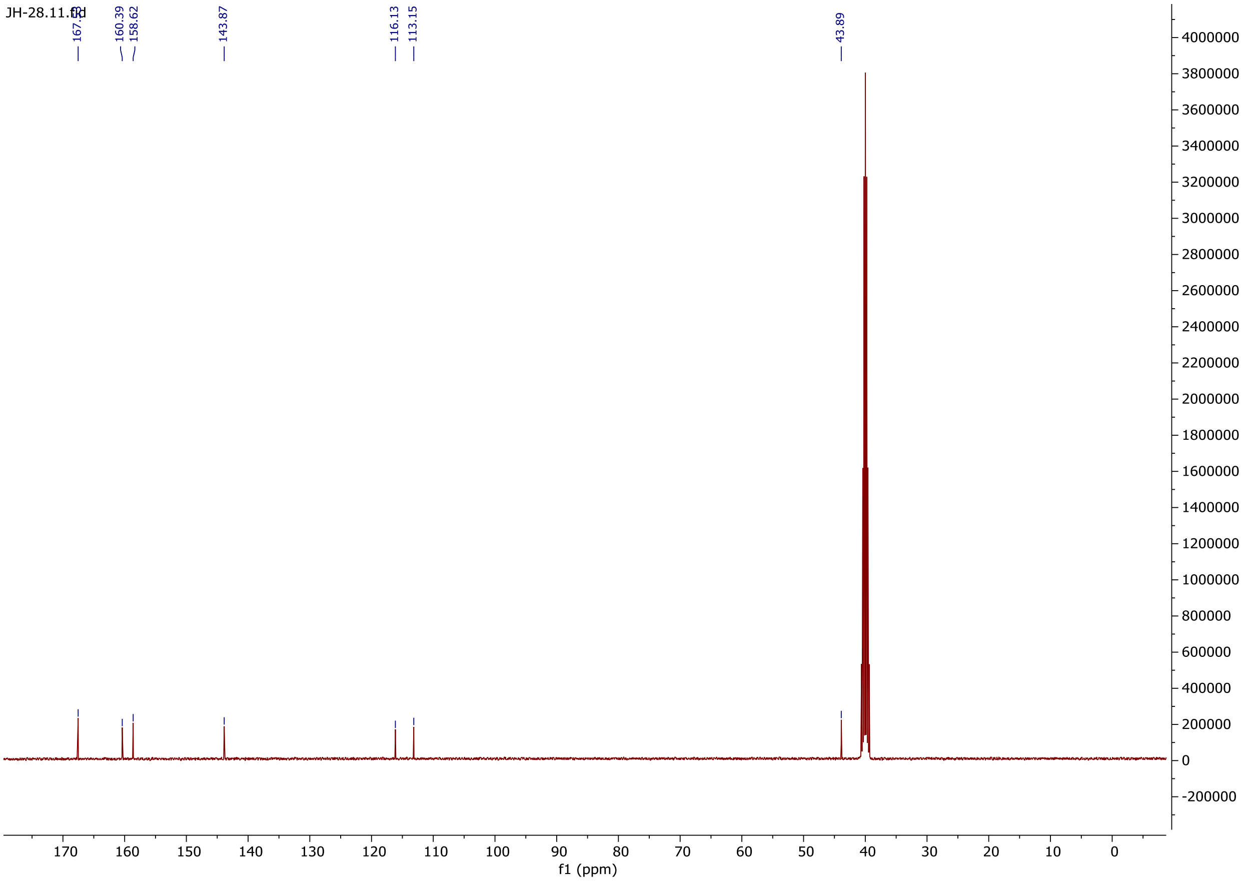

Figure S14: ^{13}C NMR for mCAmCP

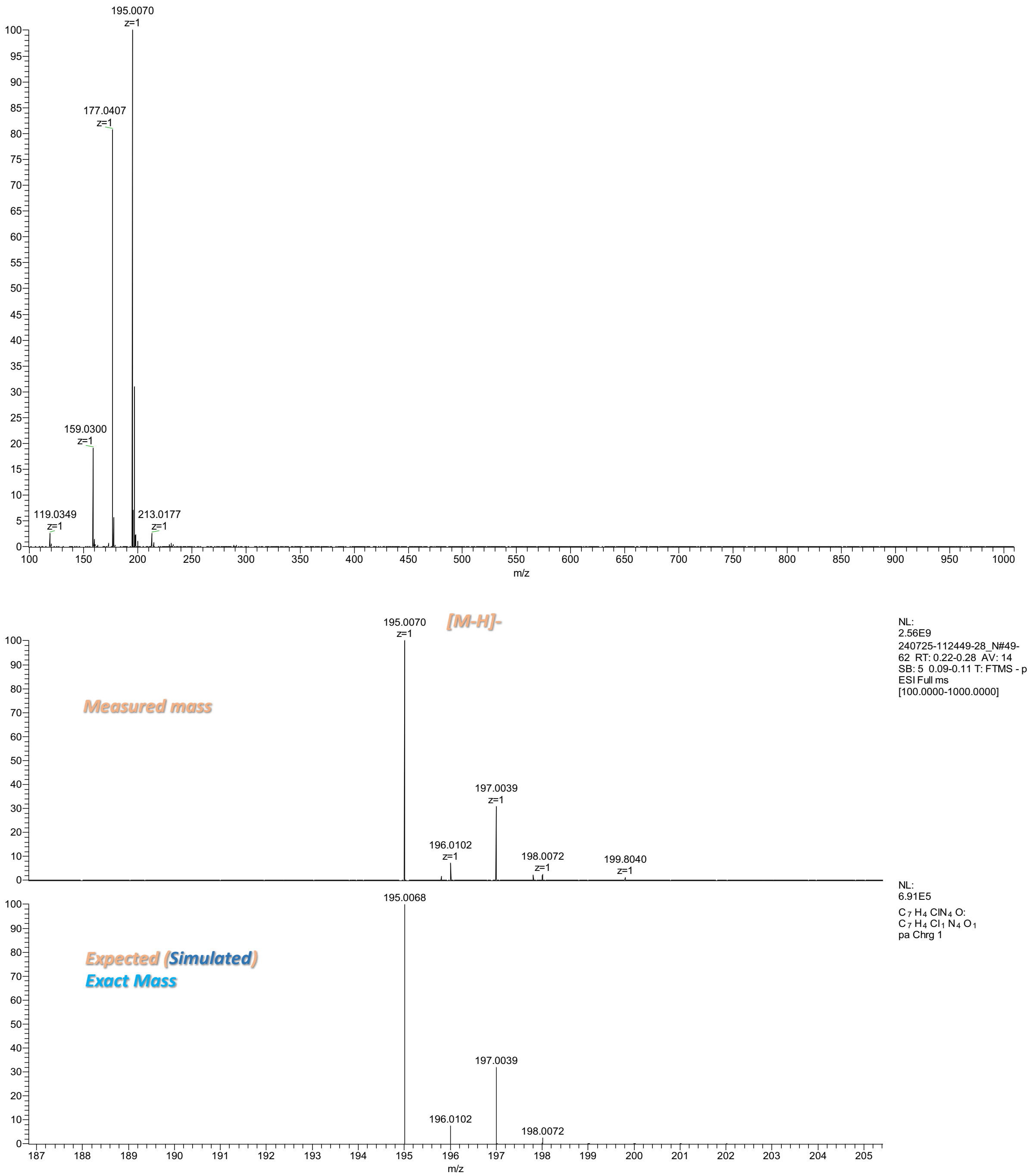

Figure S15: HRMS data for mCAmCP. The top spectra corresponds to the TIC from a direct injection ranging from 100-1000 (m/z) in negative mode (ESI). The bottom gives a comparison between the observed and expected mass isotope distribution.

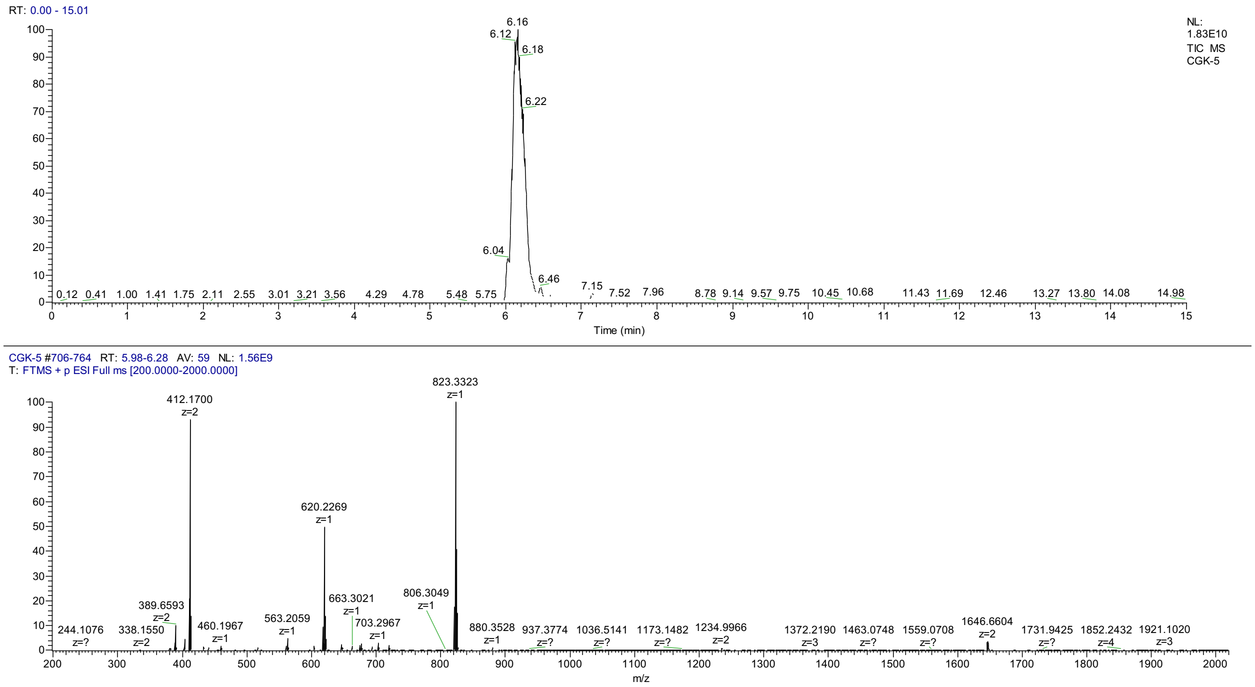

Figure S16: LC-MS Data for CGK-5. The TIC chromatogram is shown on top and the extracted masses from the peak are below.

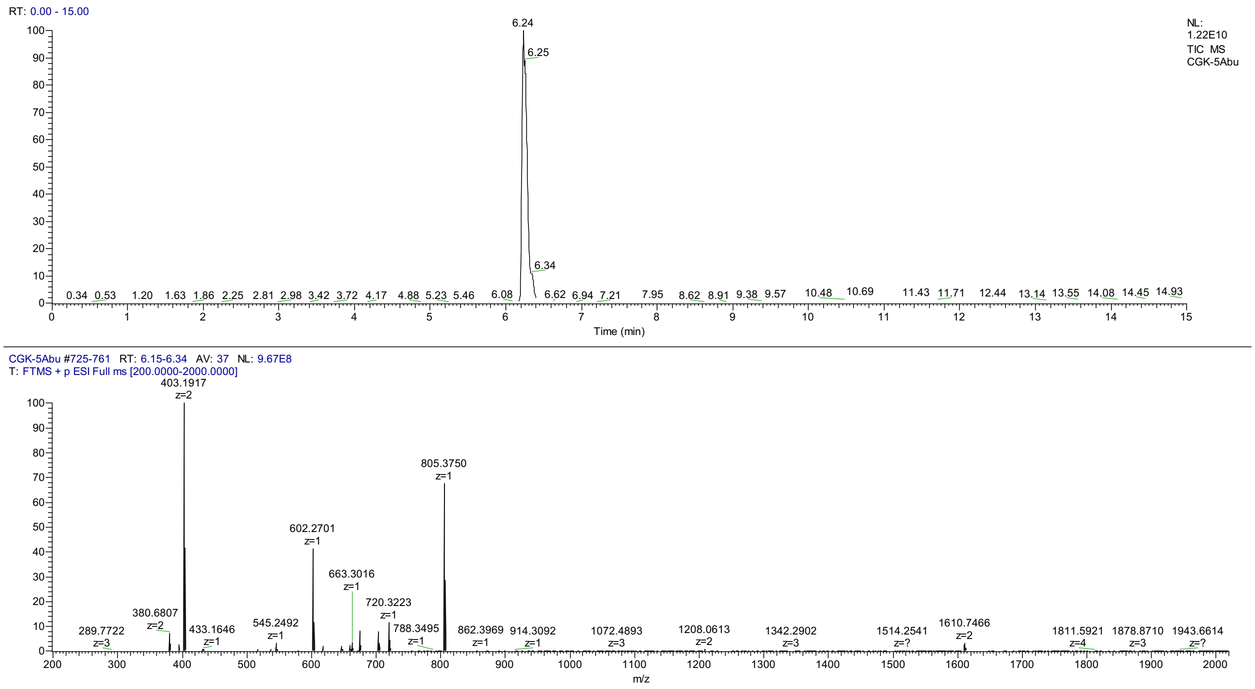

Figure S17: LC-MS Data for CGK-5Abu. The TIC chromatogram is shown on top and the extracted masses from the peak are below.

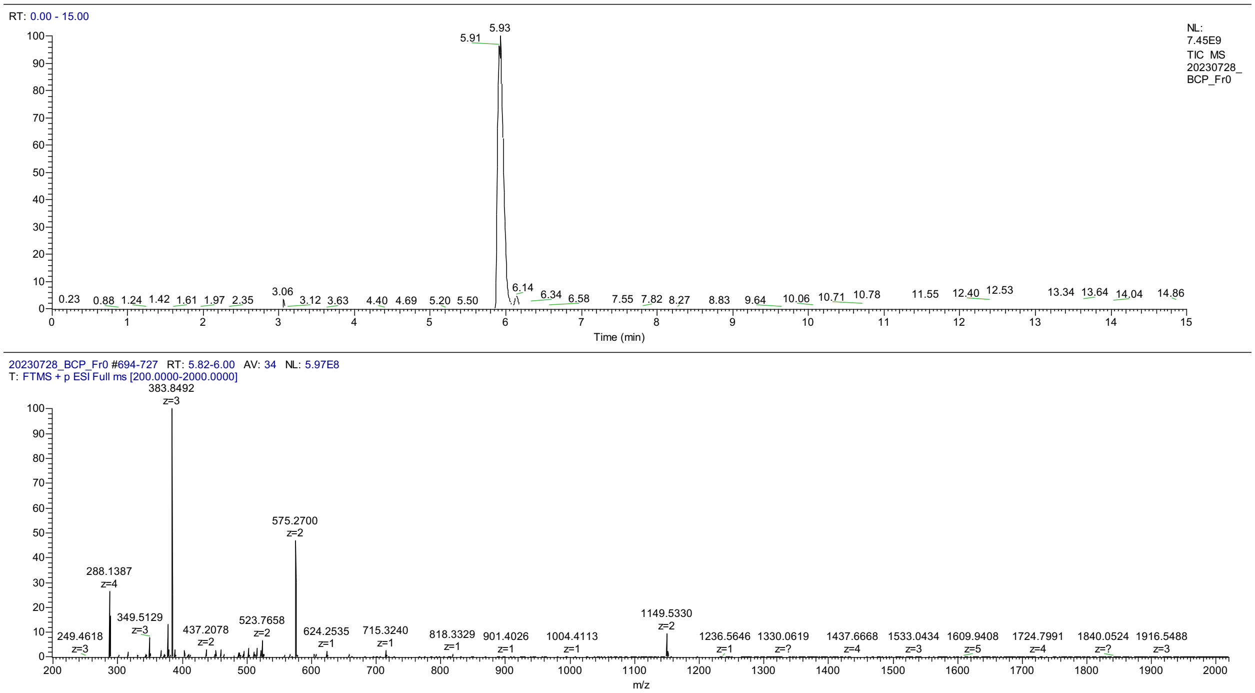

Figure S18: LC-MS Data for BCP. The TIC chromatogram is shown on top and the extracted masses from the peak are below.

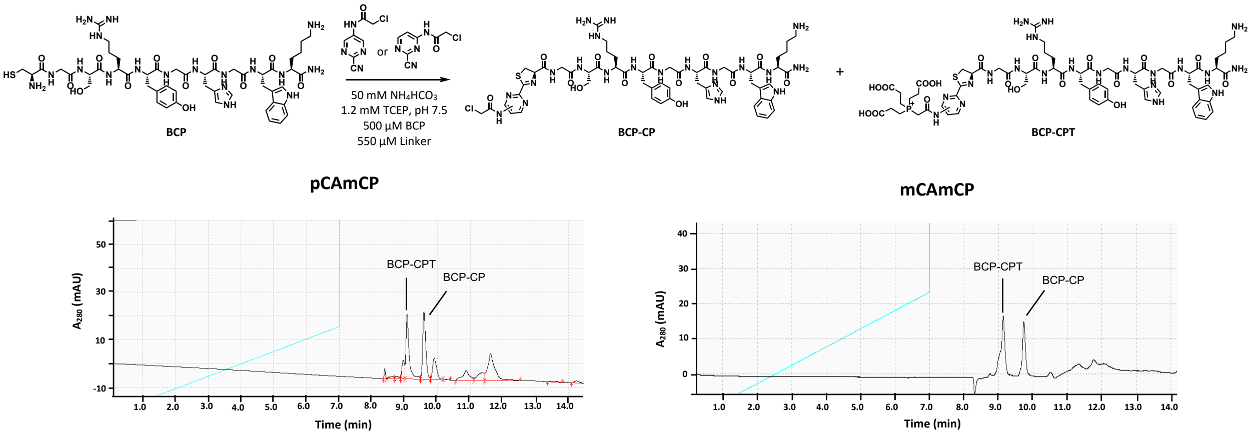

Figure S19: Reaction between a peptide (BCP) containing nucleophilic residues other than cysteine and CAmCP linkers. Representative HPLC traces after four hours of reaction are given. Masses corresponding to BCP-CP and BCP-CPT were observed in the spectra and their peaks are labelled on each chromatogram.

A

SKETAFVEVVLFESSPSGDYTTYTTGLTGRFSRAGATLSAEGEIVQMHPLGLCNNNDEEDLYEYG
WVGVVKLEQPELDPKPCLTVLGKAKRAVQRGATAVIFDVSENPEAIDQLNQGSEDPLKRPVVYV
KGADAIKLMNIVNKQKVARARIQHRPPRQPTEYFDMGLNDIFEAQKIEWHE

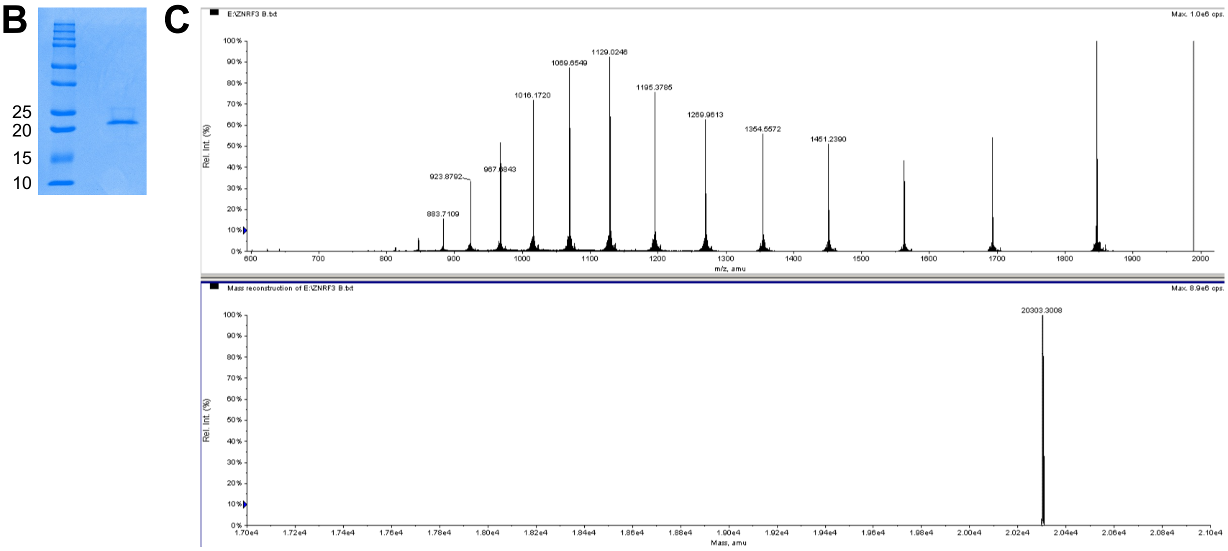

Figure S20: Characterization of biotinylated ZNRF3 extracellular domain. A) Protein sequence for the extracellular domain of ZNRF3 (black) fused to the AviTag for biotinylation by BirA (red). B) SDS-PAGE analysis of purified sample. Molecular weight standards are labelled in KDa. C) High resolution mass spectrometry of the purified sample and deconvolution of the data. The observed molecular weight matched the expected molecular weight (20303.3 Da). The protein used was the same as previously published.[1]

A

Round 4 Sequences

	V1	V2	V3	V4	V5	V6	V7	V8	V9	V10	V11	V12	V13	V14	n	percent
**Z27S1**	C	H	L	S	P	L	E	A	W	R	Q	K	L	C	46634	67.1969337
**Z27S2**	C	D	V	Q	F	W	L	S	L	D	R	L	R	C	7010	10.1010101
	C	W	P	E	W	M	H	P	Q	F	D	T	S	C	4630	6.67156587
	C	V	C	V	W	C	C	V	V	Y	H	F	TAG	C	3438	4.95396187
**Z27S3**	C	P	N	A	A	TAG	F	I	R	Y	S	R	A	C	1324	1.90780847
	C	P	P	G	W	W	R	G	H	P	Q	F	P	C	383	0.55188115
	C	L	F	R	S	R	V	R	M	TAG	W	N	G	C	333	0.479834
	C	G	G	G	W	D	R	F	R	H	L	G	W	C	328	0.47262929
	C	V	R	T	A	E	L	I	Y	C	TAG	V	S	C	178	0.25648785
	C	A	R	P	C	R	W	S	L	E	R	V	S	C	166	0.23919653
	C	P	K	L	A	N	Y	Y	E	I	E	Y	W	C	111	0.15994467
	C	V	C	T	K	F	M	S	K	F	F	C	W	C	93	0.13400769
	C	C	TAG	N	F	TAG	E	Y	N	S	W	L	G	C	83	0.11959827
	C	V	G	V	P	P	G	T	L	E	F	A	M	C	48	0.06916526
	C	T	F	M	F	R	A	H	P	Q	F	E	T	C	39	0.05619678

B

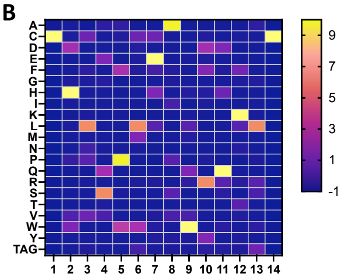

C

Round 4 vs Round 1 Comparison

	V1	V2	V3	V4	V5	V6	V7	V8	V9	V10	V11	V12	V13	V14	n	percent	log2foldchange
**Z27S2**	C	D	V	Q	F	W	L	S	L	D	R	L	R	C	7010	10.1010101	10.47325477
	C	L	F	R	S	R	V	R	M	TAG	W	N	G	C	333	0.479834003	8.3993625
	C	A	R	P	C	R	W	S	L	E	R	V	S	C	166	0.23919653	7.395023564
	C	L	I	E	G	W	W	Y	V	E	Q	S	S	C	13	0.018732258	3.720423851
	C	A	H	Q	S	K	Y	TAG	L	I	G	TAG	N	C	11	0.015850372	3.479415751
	C	A	N	C	F	Y	V	V	W	N	R	V	L	C	11	0.015850372	3.479415751
	C	M	M	L	L	S	T	N	TAG	T	K	V	D	C	7	0.010086601	2.827339055
	C	Q	S	V	Q	L	N	W	K	G	G	K	G	C	7	0.010086601	2.827339055
	C	G	TAG	Q	R	I	D	R	Y	L	A	G	K	C	5	0.007204715	2.341912227
	C	L	I	C	P	S	F	R	S	F	S	K	S	C	5	0.007204715	2.341912227
	C	S	TAG	F	S	W	S	A	N	I	G	G	T	C	4	0.005763772	2.019984132
	C	T	L	W	C	V	V	H	A	S	W	R	V	C	4	0.005763772	2.019984132
	C	T	T	V	L	TAG	K	TAG	R	L	V	K	A	C	4	0.005763772	2.019984132
	C	D	TAG	I	K	L	Y	S	F	N	R	S	H	C	3	0.004322829	1.604946633

D

Round 4 vs Round 2 Comparison

	V1	V2	V3	V4	V5	V6	V7	V8	V9	V10	V11	V12	V13	V14	n	percent	log2foldchange
**Z27S1**	C	H	L	S	P	L	E	A	W	R	Q	K	L	C	46634	67.19693367	14.73652974
**Z27S2**	C	D	V	Q	F	W	L	S	L	D	R	L	R	C	7010	10.1010101	13.00263391
**Z27S3**	C	P	N	A	A	TAG	F	I	R	Y	S	R	A	C	1324	1.90780847	9.598122589
	C	V	C	V	W	C	C	V	V	Y	H	F	TAG	C	3438	4.953961873	8.974789012
	C	A	R	P	C	R	W	S	L	E	R	V	S	C	166	0.23919653	6.602474613
	C	C	TAG	N	F	TAG	E	Y	N	S	W	L	G	C	83	0.119598265	6.602474613
	C	G	G	G	W	D	R	F	R	H	L	G	W	C	328	0.472629289	6.584987187
	C	L	F	R	S	R	V	R	M	TAG	W	N	G	C	333	0.479834003	5.147381931
	C	G	V	T	F	H	S	L	L	P	I	S	I	C	20	0.028818859	4.549363277
	C	V	C	T	K	F	M	S	K	F	F	C	W	C	93	0.134007695	3.959239071
	C	K	P	F	R	F	W	L	F	H	Y	D	T	C	13	0.018732258	3.9278749
	C	L	I	E	G	W	W	Y	V	E	Q	S	S	C	13	0.018732258	3.9278749
	C	S	L	R	R	F	G	TAG	A	W	R	H	V	C	24	0.034582631	3.812397683
	C	E	V	H	P	Q	F	P	F	P	T	Y	G	C	12	0.017291315	3.812397683

Figure S21: Summary of next-generation sequencing of pCAmCP cyclized phages selected against ZNRF3-ECD. A) Most abundant sequences after four rounds of selection. Peptides chosen for characterization are labelled. TAG = glutamine in amber-suppressing cell line ER2738. B) Heat map of amino acid abundance at each position in the phage library after four rounds of selection. Abundance was calculated as the bias from a true NNK library (%R4 - %randomized)/%randomized. Differential enrichment analysis was performed for sequences that were found in rounds 4 and 1 of selection (C) and rounds 4 and 2 of selection (D). Log2foldchange was calculated by Log_2(%final/%initial).

A

Round 4 Sequences

	V1	V2	V3	V4	V5	V6	V7	V8	V9	V10	V11	V12	V13	V14	n	percent
**Z28S1**	C	F	L	R	L	N	W	E	N	L	M	P	W	C	68432	81.37657118
**Z28S2**	C	E	L	V	M	M	H	G	Y	W	W	R	Q	C	7379	8.774808843
	C	R	V	TAG	F	Y	Y	L	C	M	Y	F	R	C	535	0.636200397
	C	Y	G	W	V	I	H	R	T	H	E	I	A	C	279	0.331775534
	C	F	W	S	A	T	S	T	G	C	R	S	R	C	186	0.221183689
	C	V	Y	I	TAG	V	G	TAG	L	TAG	A	T	G	C	159	0.18907638
	C	D	F	D	F	A	L	V	C	TAG	K	F	Y	C	116	0.137942516
	C	L	L	R	W	L	N	A	R	TAG	C	P	S	C	61	0.072538737
	C	H	A	C	S	A	L	S	G	L	Q	R	S	C	58	0.068971258
	C	P	A	TAG	Q	I	F	L	T	S	G	N	L	C	57	0.067782098
	C	F	L	R	L	N	W	E	N	L	M	S	W	C	55	0.065403779
	C	L	S	Y	I	P	V	T	A	P	R	TAG	Y	C	44	0.052323023
	C	S	H	TAG	I	E	S	V	TAG	E	T	C	K	C	36	0.042809746
	C	D	K	S	R	A	C	N	Y	C	S	F	W	C	29	0.034485629

B

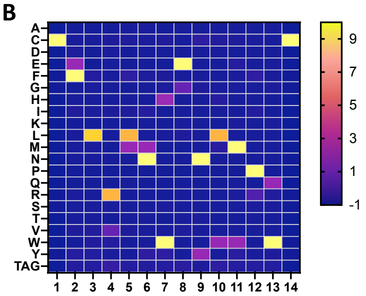

C

Round 4 vs Round 1 Comparison

	V1	V2	V3	V4	V5	V6	V7	V8	V9	V10	V11	V12	V13	V14	n	log2foldchange
**Z28S1**	C	F	L	R	L	N	W	E	N	L	M	P	W	C	68432	15.7816284
	C	L	L	R	W	L	N	A	R	TAG	C	P	S	C	61	4.649982249
	C	S	R	TAG	L	P	S	TAG	K	V	M	L	F	C	14	3.526599833
	C	I	N	F	I	A	Y	S	F	V	E	T	TAG	C	11	3.17867653
	C	E	S	S	S	K	TAG	R	S	L	D	Y	K	C	9	2.889169913
	C	R	Q	TAG	E	S	K	F	A	A	R	T	V	C	9	2.889169913
	C	S	Y	V	A	V	S	V	E	L	R	Y	TAG	C	8	2.719244911
	C	V	S	R	S	I	G	M	V	P	F	S	TAG	C	8	2.719244911
	C	Y	T	R	V	TAG	W	L	S	G	H	V	K	C	8	2.719244911
	C	A	P	L	P	L	W	TAG	L	D	L	R	K	C	14	2.526599833
	C	A	Y	P	S	W	TAG	L	A	T	V	D	E	C	7	2.526599833
	C	C	R	TAG	T	G	C	M	A	M	T	T	W	C	7	2.526599833
	C	M	G	TAG	G	S	C	V	N	V	V	K	V	C	7	2.526599833
	C	M	R	L	L	I	TAG	T	D	N	T	D	D	C	7	2.526599833

D

Round 4 vs Round 2 Comparison

	V1	V2	V3	V4	V5	V6	V7	V8	V9	V10	V11	V12	V13	V14	n	percent	log2foldchange
**Z28S2**	C	E	L	V	M	M	H	G	Y	W	W	R	Q	C	7379	8.774808843	12.3857966
	C	R	V	TAG	F	Y	Y	L	C	M	Y	F	R	C	535	0.636200397	8.599982083
	C	Y	G	W	V	I	H	R	T	H	E	I	A	C	279	0.331775534	5.075745813
	C	F	TAG	S	C	I	Y	N	L	N	F	L	F	C	22	0.026161512	3.996018621
	C	H	A	C	S	A	L	S	G	L	Q	R	S	C	58	0.068971258	3.809605496
	C	F	W	S	A	T	S	T	G	C	R	S	R	C	186	0.221183689	3.753817718
	C	V	M	R	D	A	G	T	Q	A	V	W	A	C	18	0.021404873	3.706512003
	C	L	S	Y	I	P	V	T	A	P	R	TAG	Y	C	44	0.052323023	3.41105612
	C	P	A	TAG	Q	I	F	L	T	S	G	N	L	C	57	0.067782098	3.369477016
	C	T	S	S	Y	TAG	A	Y	F	R	G	R	L	C	14	0.016648235	3.343941924
	C	L	L	R	S	C	D	R	F	I	E	G	W	C	13	0.015459075	3.23702672
	C	V	L	E	Q	G	TAG	F	S	K	V	M	P	C	13	0.015459075	3.23702672
	C	H	L	S	P	L	E	A	W	R	Q	K	L	C	12	0.014269915	3.121549503
	C	V	R	M	V	H	I	G	E	T	C	V	W	C	12	0.014269915	3.121549503

Figure S22: Summary of next-generation sequencing of mCAmCP cyclized phages selected against ZNRF3-ECD. A) Most abundant sequences after four rounds of selection. TAG = glutamine in amber-supressing cell line ER2738. Peptides chosen for characterization are labelled. B) Heat map of amino acid abundance at each position in the phage library after four rounds of selection. Abundance was calculated as the bias from a true NNK library (%R4 - %randomized)/%randomized. Differential enrichment analysis was performed for sequences that were found in rounds 4 and 1 of selection (C) and rounds 4 and 2 of selection (D). Log2foldchange was calculated by Log_2(%final/%initial).

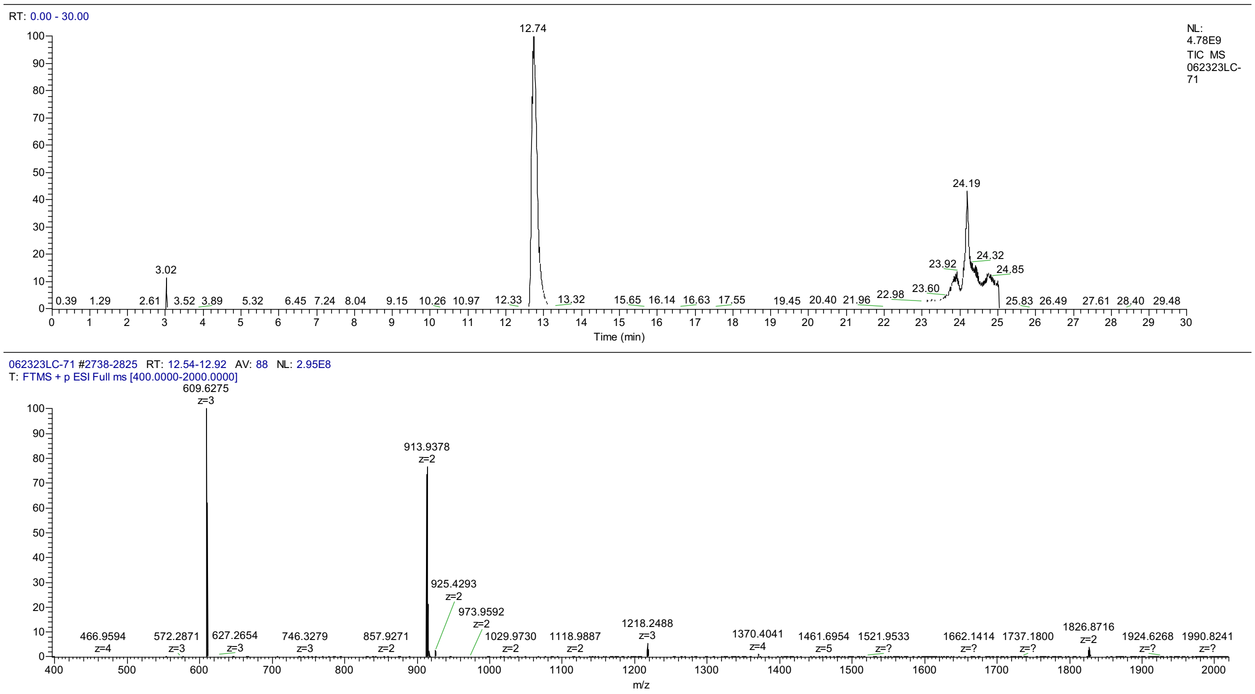

Figure S23: LC-MS Data for Z27S1. The TIC chromatogram is shown on top and the extracted masses from the peak are below.

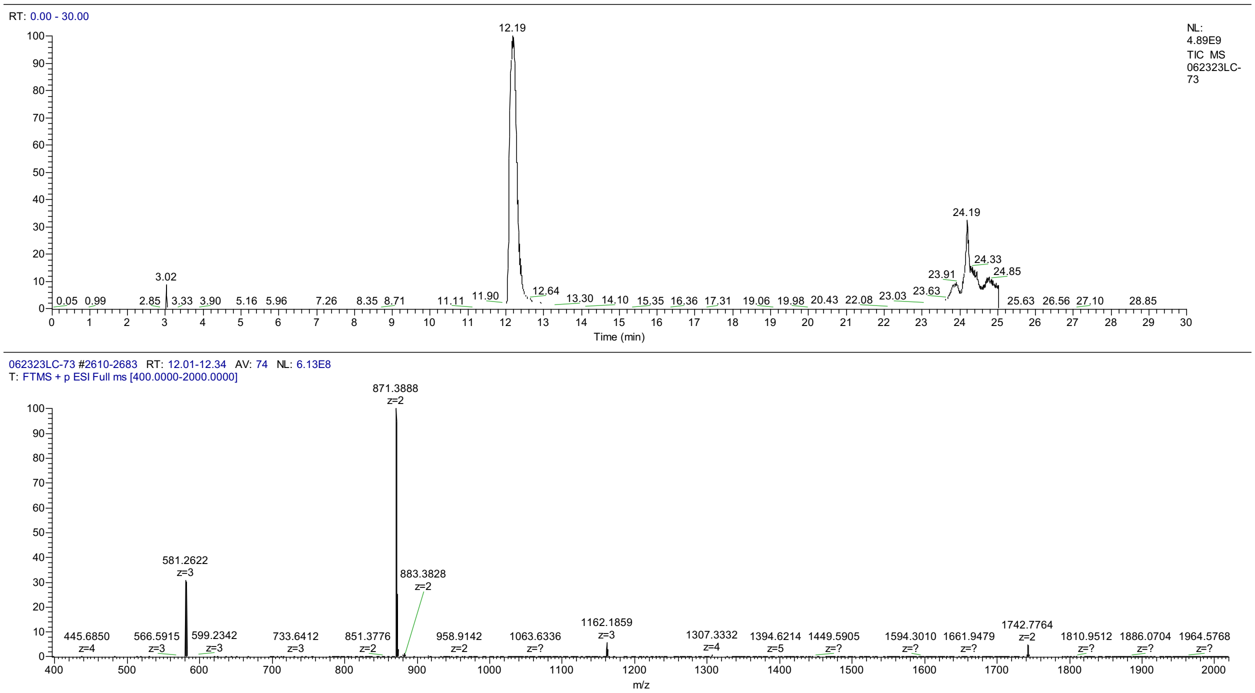

Figure S24: LC-MS Data for Z27S3. The TIC chromatogram is shown on top and the extracted masses from the peak are below.

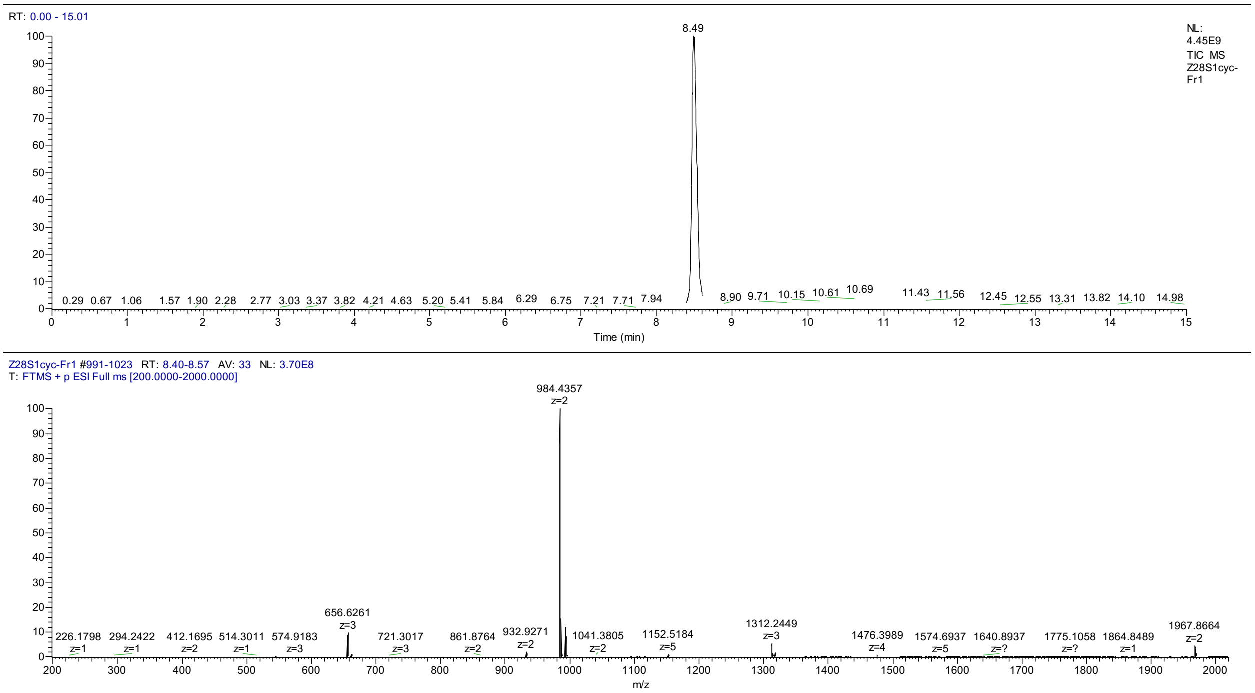

Figure S25: LC-MS Data for Z28S1. The TIC chromatogram is shown on top and the extracted masses from the peak are below.

Peptide	Peptide Sequence	K_D (μM)
Z28S1	C F L R L N W E N L M P W C	>40
Z28S2	C E L V M M H G Y W W R Q C	n.t.

C = mCAmCP reacted, a = Abu
n.t. not tested, poor solubility during synthesis
n.d. not determined

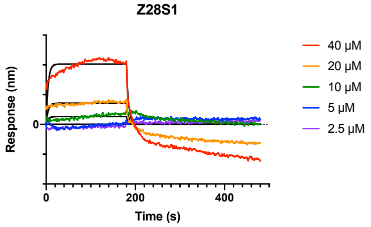

Figure S26: Biolayer interferometry assays for Z28S1 binding to ZNRF3-ECD showed high off rates resulting in a K_D greater than 40 μM.

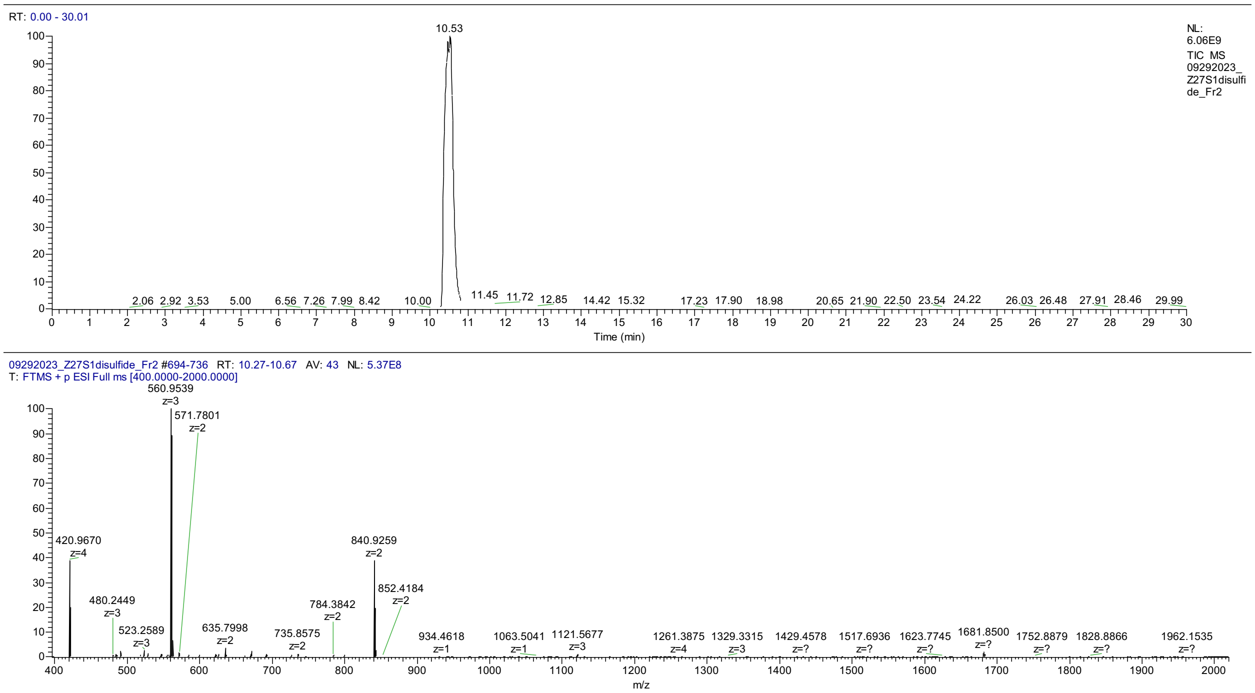

Figure S27: LC-MS Data for Z27S1Disulf. The TIC chromatogram is shown on top and the extracted masses from the peak are below.

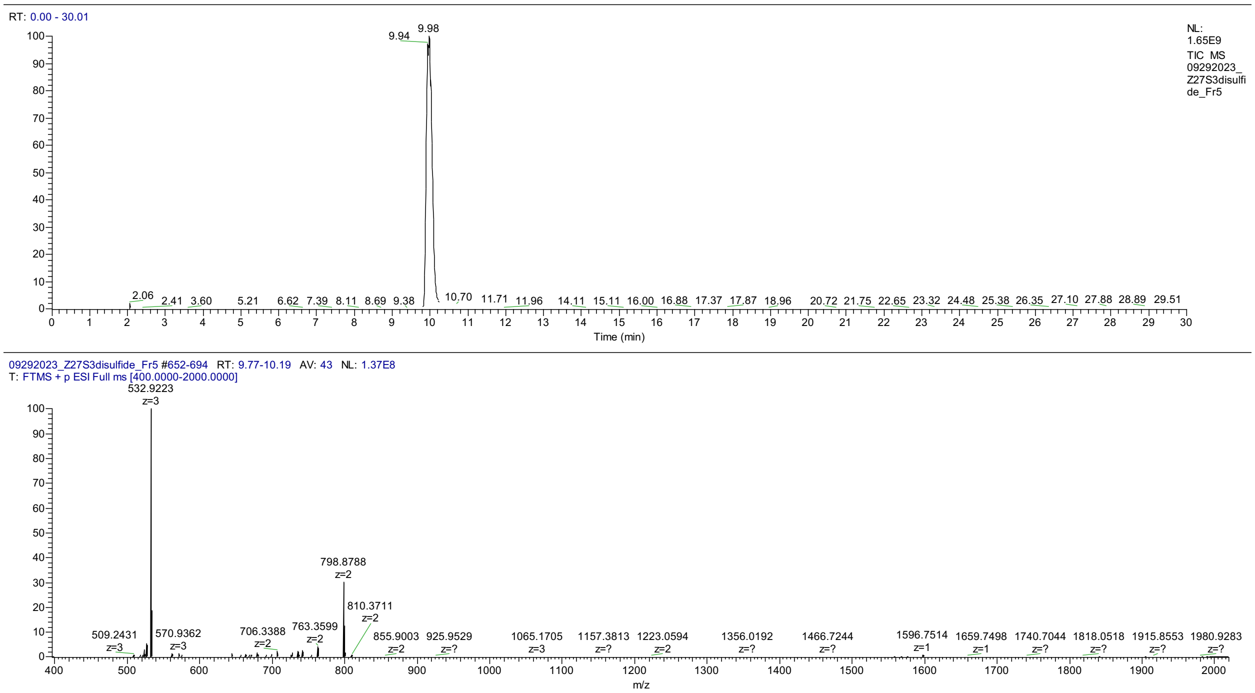

Figure S28: LC-MS Data for Z27S3Disulf. The TIC chromatogram is shown on top and the extracted masses from the peak are below.

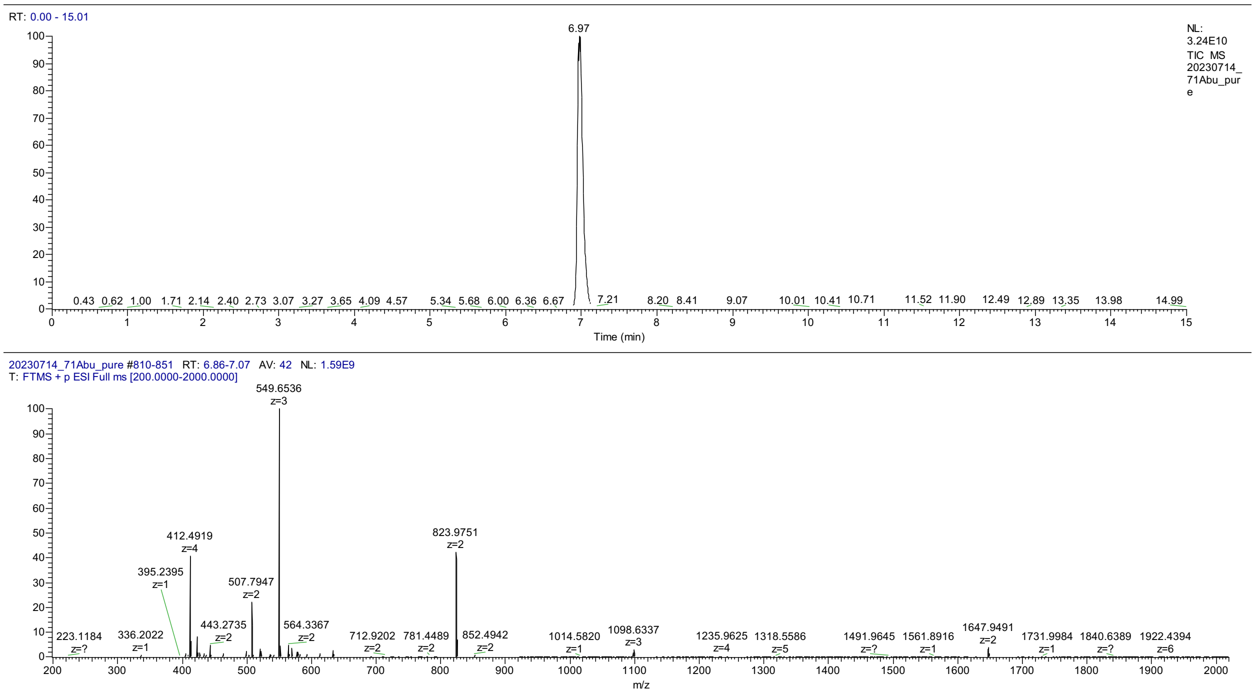

Figure S29: LC-MS Data for Z27S1Abu. The TIC chromatogram is shown on top and the extracted masses from the peak are below.

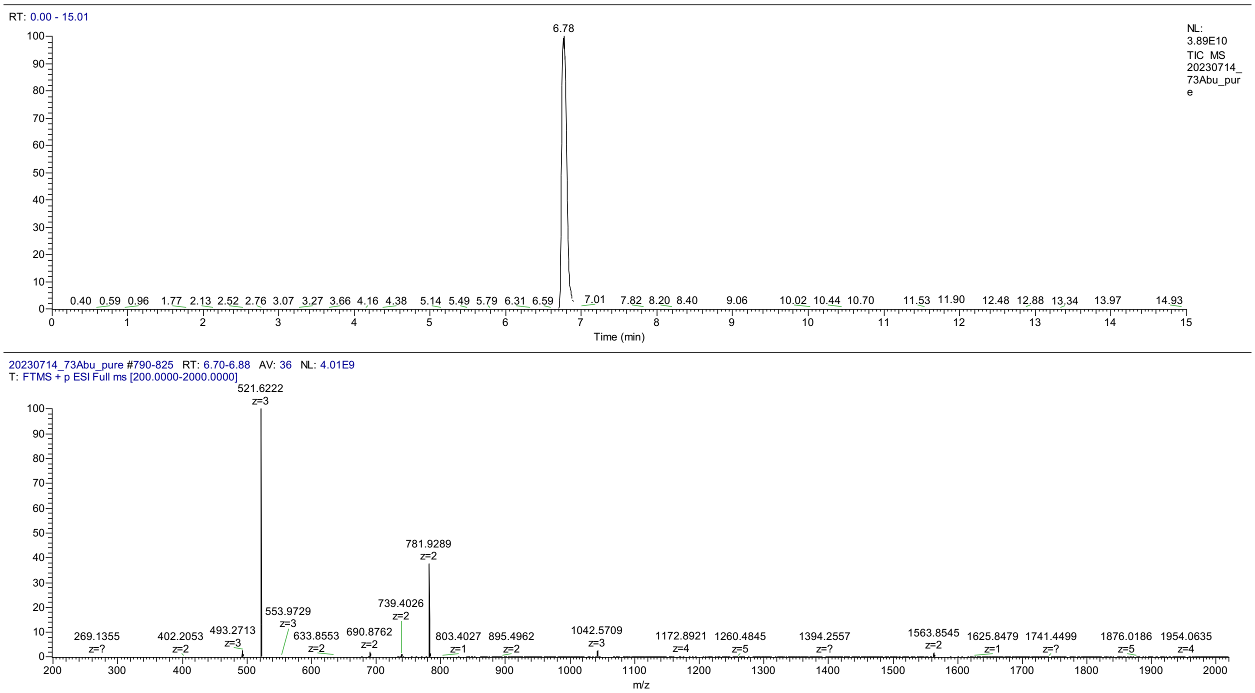

Figure S30: LC-MS Data for Z27S3Abu. The TIC chromatogram is shown on top and the extracted masses from the peak are below.

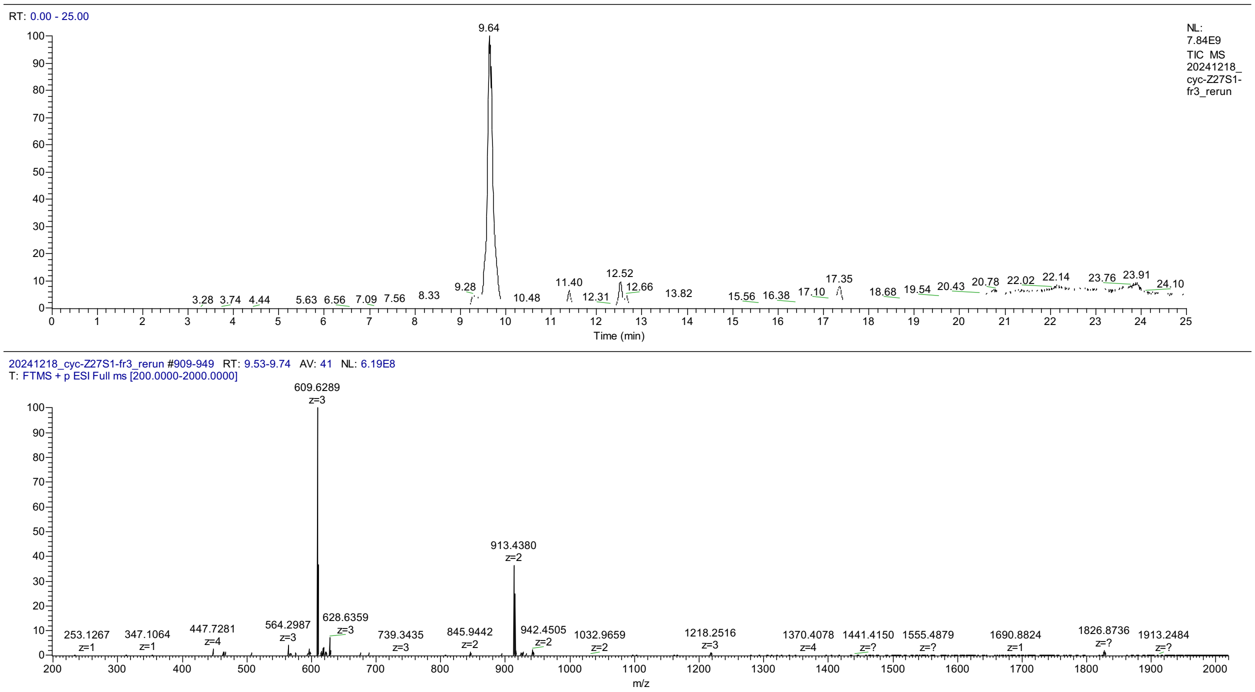

Figure S31: LC-MS Data for Z27S1-mCP. The TIC chromatogram is shown on top and the extracted masses from the peak are below.

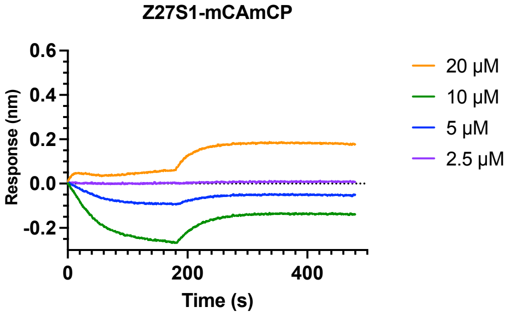

Figure S32: Biolayer Interferometry Experiments for Z27S1-mCP interacting with immobilized biotinylated ZNRF3-ECD. No significant dose-dependent interactions were observed.

SUPPLEMENTARY TABLES

Table S1: High-Resolution Mass Spectrometry Data for Synthesized Peptides

Peptide	Sequence	Linker	Expected Mass (Da)	Observed Mass (Da)
CGK-5	CGGKGGCGW	None	822.33	822.33
CGK-5Abu	AbuGGKGGCGW	None	804.37	804.37
BCP	CGSRYGHGWK	None	1148.53	1148.53
Z27S1	CHLSPLEAWRQKLC	pCAmCP	1824.87	1824.87
Z27S3	CPNAAQFIRYSRAC	pCAmCP	1740.77	1740.78
Z28S1	CFLRLNWENLMPWC	mCAmCP	1965.86	1965.87
Z27S1Disulf	CHLSPLEAWRQKLC	Disulfide	1679.84	1679.85
Z27S1Abu	AbuHLSPLEAWRQKLAbu	None	1645.94	1645.94
Z27S3Disulf	CPNAAQFIRYSRAC	Disulfide	1595.74	1595.75
Z27S3Abu	AbuPNAAQFIRYSRAAbu	None	1561.85	1561.86
Z27S1-mCP	CHLSPLEAWRQKLC	mCAmCP	1824.87	1824.88

Abu: Aminobutyric acid

Table S2: HPLC-MS Purification Conditions for peptides

Peptide	Gradient*	Column	Ionization Mode	Flow Rate (mL/min)
CGK-5	0-20%ACN in water over 30 min	C18, 250x20 mm	ESI (+)	15
CGK-5Abu	0-20%ACN in water over 30 min	C18, 250x20 mm	ESI (+)	15
BCP	0-20%ACN in water over 30 min	C18, 250x20 mm	ESI (+)	15
Z27S1	10-25%ACN in water over 30 min	C18, 250x20 mm	ESI (+)	15
Z27S3	10-25%ACN in water over 30 min	C18, 250x20 mm	ESI (+)	15
Z28S1	30-40%ACN in water over 30 min	C18, 250x20 mm	ESI (+)	15
Z27S1Disulf	10-25%ACN in water over 30 min	C18, 250x20 mm	ESI (+)	15
Z27S1Abu	10-40%ACN in water over 30 min	C18, 250x20 mm	ESI (+)	15
Z27S3Disulf	10-25%ACN in water over 30 min	C18, 250x20 mm	ESI (+)	15
Z27S3Abu	10-20%ACN in water over 30 min	C18, 250x20 mm	ESI (+)	15
Z27S1-mCP	10-25%ACN in water over 30 min	C18, 250x20 mm	ESI (+)	15

*All solvents contained 0.1% formic acid as additive

SUPPLEMENTARY SCRIPTS

Supplementary Script 1: Amino Acid Analysis

```
library(microseq)
library(RColorBrewer)
library(dplyr)
library(stringr)
library(gplots)
NNK7Ffilt <-
 readFastq("/Users/traehampton/Documents/Research/Sequencing
 Results/Next Gen
 Sequencing/20093Wns_N20056/20093Wns_P28_12mer_R3_S2_L001_R1_001.fastq"
 )
NNK7Rfilt <-
 readFastq("/Users/traehampton/Documents/Research/Sequencing
 Results/Next Gen
 Sequencing/23381Wns_N23171/23381Wns_R3ENL_S2_L001_R2_001.fastq")
#Define the following variables
libraryseq <- "GCCCAG.{54}GCGGCG.{6}" #change this regex to match
 specific library
beginning <- 19 #beginning of library in DNA string
lib <- 14 #number of codons in the library region
end <- beginning+lib*3
initialcodon <- beginning%/%3*4-1
endcodon <- initialcodon + lib*4
del <- beginning%/%3 #number of codons before library
aa <-
 c("A","C","D","E","F","G","H","I","K","L","M","N","P","Q","R","S","T",
 "V","W","Y","TAG")

#slices out matches that contain start followed by 24 bases to reverse
 primer
NNK7Ffilt21 <- gregexpr(libraryseq,NNK7Ffilt[[2]],extract = TRUE)
NNK7Rrevcomp <- reverseComplement(NNK7Rfilt[[2]],reverse = TRUE)
 #gives reverse complement of reverse reads
NNK7Rcompfilt21 <- gregexpr(libraryseq,NNK7Rrevcomp,extract = TRUE)

#this compares the forward and reverse strands, only allowing for one
 mismatch in the primers, no mismatches allowed in the library region
n <- length(NNK7Ffilt21)
NNK7Fgood <- vector()
for(i in c(1:n)){
  if(NNK7Ffilt21[[i]][1] == NNK7Rcompfilt21[[i]][1]){
    NNK7Fgood[i] <- NNK7Ffilt21[[i]]
  }
```

```
  else{
    split <- strsplit(c(NNK7Ffilt21[[i]],NNK7Rcompfilt21[[i]]), split
 = "")
    diff <- which(split[[1]] != split[[2]])
    if(length(diff) < 2 && length(diff) > 0){
      for(x in c(1:length(diff))){
        if(diff[[x]] < beginning || diff[[x]] > end){
          NNK7Fgood[i] <- NNK7Ffilt21[[i]]
        }
        else{
          NNK7Fgood[i] <- ""
        }
      }
    }
    else{
      NNK7Fgood[i] <- ""
    }
  }
}
NNK7Fgood <- as.data.frame(NNK7Fgood)
NNK7Fgood <- NNK7Fgood[!apply(is.na(NNK7Fgood) | NNK7Fgood == "", 1,
 all),]

#this separates nucleotides into codons
codons <- gsub("(...)", "\\1 \\2", NNK7Fgood)

#this creates dataframe of sequences with reads organized by frequency
seqcount <- as.data.frame(sort(table(codons), decreasing = TRUE))

#this generates a matrix that contains amino acids in library region
l <- length(codons)
AAs <- matrix(0,l,lib)
AA <- gregexpr("\\s(TT[TC])",codons,useBytes = FALSE)
  l <- length(AA)
  for(a in c(1:l)){
    l2 <- length(AA[[a]])
    for(b in c(1:l2)){
      value <- AA[[a]][b]
      if(value > initialcodon && value < endcodon){
        AAs[a,(value%/%4 - (del-1))] <- "F"
      }
    }
  }
AA <- gregexpr("(\\sTT[AG])|(\\sCT[GACT])",codons,useBytes = FALSE)
```

```
  l <- length(AA)
  for(a in c(1:l)){
    l2 <- length(AA[[a]])
    for(b in c(1:l2)){
      value <- AA[[a]][b]
      if(value > initialcodon && value < endcodon){
        AAs[a,(value%/%4 - (del-1))] <- "L"
      }
    }
  }
AA <- gregexpr("(\\sTC[GCAT])|(\\sAG[TC])",codons,useBytes = FALSE)
  l <- length(AA)
  for(a in c(1:l)){
    l2 <- length(AA[[a]])
    for(b in c(1:l2)){
      value <- AA[[a]][b]
      if(value > initialcodon && value < endcodon){
        AAs[a,(value%/%4 - (del-1))] <- "S"
      }
    }
  }
AA <- gregexpr("\\sTA[TC]",codons,useBytes = FALSE)
  l <- length(AA)
  for(a in c(1:l)){
    l2 <- length(AA[[a]])
    for(b in c(1:l2)){
      value <- AA[[a]][b]
      if(value > initialcodon && value < endcodon){
        AAs[a,(value%/%4 - (del-1))] <- "Y"
      }
    }
  }
AA <- gregexpr("\\sTAG",codons,useBytes = FALSE)
  l <- length(AA)
  for(a in c(1:l)){
    l2 <- length(AA[[a]])
    for(b in c(1:l2)){
      value <- AA[[a]][b]
      if(value > initialcodon && value < endcodon){
        AAs[a,(value%/%4 - (del-1))] <- "TAG"
      }
    }
  }
AA <- gregexpr("\\sTAA",codons,useBytes = FALSE)
```

```
  l <- length(AA)
  for(a in c(1:l)){
    l2 <- length(AA[[a]])
    for(b in c(1:l2)){
      value <- AA[[a]][b]
      if(value > initialcodon && value < endcodon){
        AAs[a,(value%/%4 - (del-1))] <- NA
      }
    }
  }
AA <- gregexpr("\\sTG[TC]",codons,useBytes = FALSE)
  l <- length(AA)
  for(a in c(1:l)){
    l2 <- length(AA[[a]])
    for(b in c(1:l2)){
      value <- AA[[a]][b]
      if(value > initialcodon && value < endcodon){
        AAs[a,(value%/%4 - (del-1))] <- "C"
      }
    }
  }
AA <- gregexpr("\\sTGA",codons,useBytes = FALSE)
  l <- length(AA)
  for(a in c(1:l)){
    l2 <- length(AA[[a]])
    for(b in c(1:l2)){
      value <- AA[[a]][b]
      if(value > initialcodon && value < endcodon){
        AAs[a,(value%/%4 - (del-1))] <- NA
      }
    }
  }
AA <- gregexpr("\\sTGG",codons,useBytes = FALSE)
  l <- length(AA)
  for(a in c(1:l)){
    l2 <- length(AA[[a]])
    for(b in c(1:l2)){
      value <- AA[[a]][b]
      if(value > initialcodon && value < endcodon){
        AAs[a,(value%/%4 - (del-1))] <- "W"
      }
    }
  }
AA <- gregexpr("\\sCC[GCAT]",codons,useBytes = FALSE)
```

```
  l <- length(AA)
  for(a in c(1:l)){
    l2 <- length(AA[[a]])
    for(b in c(1:l2)){
      value <- AA[[a]][b]
      if(value > initialcodon && value < endcodon){
        AAs[a,(value%/%4 - (del-1))] <- "P"
      }
    }
  }
AA <- gregexpr("\\sCA[CT]",codons,useBytes = FALSE)
  l <- length(AA)
  for(a in c(1:l)){
    l2 <- length(AA[[a]])
    for(b in c(1:l2)){
      value <- AA[[a]][b]
      if(value > initialcodon && value < endcodon){
        AAs[a,(value%/%4 - (del-1))] <- "H"
      }
    }
  }
AA <- gregexpr("\\sCA[AG]",codons,useBytes = FALSE)
  l <- length(AA)
  for(a in c(1:l)){
    l2 <- length(AA[[a]])
    for(b in c(1:l2)){
      value <- AA[[a]][b]
      if(value > initialcodon && value < endcodon){
        AAs[a,(value%/%4 - (del-1))] <- "Q"
      }
    }
  }
AA <- gregexpr("(\\sCG[GCAT])|(\\sAG[GA])",codons,useBytes = FALSE)
  l <- length(AA)
  for(a in c(1:l)){
    l2 <- length(AA[[a]])
    for(b in c(1:l2)){
      value <- AA[[a]][b]
      if(value > initialcodon && value < endcodon){
        AAs[a,(value%/%4 - (del-1))] <- "R"
      }
    }
  }
AA <- gregexpr("\\sAT[CAT]",codons,useBytes = FALSE)
```

```
  l <- length(AA)
  for(a in c(1:l)){
    l2 <- length(AA[[a]])
    for(b in c(1:l2)){
      value <- AA[[a]][b]
      if(value > initialcodon && value < endcodon){
        AAs[a,(value%/%4 - (del-1))] <- "I"
      }
    }
  }
AA <- gregexpr("\\sATG",codons,useBytes = FALSE)
  l <- length(AA)
  for(a in c(1:l)){
    l2 <- length(AA[[a]])
    for(b in c(1:l2)){
      value <- AA[[a]][b]
      if(value > initialcodon && value < endcodon){
        AAs[a,(value%/%4 - (del-1))] <- "M"
      }
    }
  }
AA <- gregexpr("\\sAC[GCAT]",codons,useBytes = FALSE)
  l <- length(AA)
  for(a in c(1:l)){
    l2 <- length(AA[[a]])
    for(b in c(1:l2)){
      value <- AA[[a]][b]
      if(value > initialcodon && value < endcodon){
        AAs[a,(value%/%4 - (del-1))] <- "T"
      }
    }
  }
AA <- gregexpr("\\sAA[CT]",codons,useBytes = FALSE)
  l <- length(AA)
  for(a in c(1:l)){
    l2 <- length(AA[[a]])
    for(b in c(1:l2)){
      value <- AA[[a]][b]
      if(value > initialcodon && value < endcodon){
        AAs[a,(value%/%4 - (del-1))] <- "N"
      }
    }
  }
AA <- gregexpr("\\sAA[AG]",codons,useBytes = FALSE)
```

```
  l <- length(AA)
  for(a in c(1:l)){
    l2 <- length(AA[[a]])
    for(b in c(1:l2)){
      value <- AA[[a]][b]
      if(value > initialcodon && value < endcodon){
        AAs[a,(value%/%4 - (del-1))] <- "K"
      }
    }
  }
AA <- gregexpr("\\sGT[GACT]",codons,useBytes = FALSE)
  l <- length(AA)
  for(a in c(1:l)){
    l2 <- length(AA[[a]])
    for(b in c(1:l2)){
      value <- AA[[a]][b]
      if(value > initialcodon && value < endcodon){
        AAs[a,(value%/%4 - (del-1))] <- "V"
      }
    }
  }
AA <- gregexpr("\\sGC[GACT]",codons,useBytes = FALSE)
  l <- length(AA)
  for(a in c(1:l)){
    l2 <- length(AA[[a]])
    for(b in c(1:l2)){
      value <- AA[[a]][b]
      if(value > initialcodon && value < endcodon){
        AAs[a,(value%/%4 - (del-1))] <- "A"
      }
    }
  }
AA <- gregexpr("\\sGA[TC]",codons,useBytes = FALSE)
  l <- length(AA)
  for(a in c(1:l)){
    l2 <- length(AA[[a]])
    for(b in c(1:l2)){
      value <- AA[[a]][b]
      if(value > initialcodon && value < endcodon){
        AAs[a,(value%/%4 - (del-1))] <- "D"
      }
    }
  }
AA <- gregexpr("\\sGA[AG]",codons,useBytes = FALSE)
```

```
  l <- length(AA)
  for(a in c(1:l)){
    l2 <- length(AA[[a]])
    for(b in c(1:l2)){
      value <- AA[[a]][b]
      if(value > initialcodon && value < endcodon){
        AAs[a,(value%/%4 - (del-1))] <- "E"
      }
    }
  }
AA <- gregexpr("\\sGG[GACT]",codons,useBytes = FALSE)
  l <- length(AA)
  for(a in c(1:l)){
    l2 <- length(AA[[a]])
    for(b in c(1:l2)){
      value <- AA[[a]][b]
      if(value > initialcodon && value < endcodon){
        AAs[a,(value%/%4 - (del-1))] <- "G"
      }
    }
  }
AAs <- as.data.frame(AAs)
#this gives unique amino acid sequences
UniqueAAs <- AAs %>% group_by_all() %>% count()
UniqueAAs <- UniqueAAs[order(-UniqueAAs$n),]
UniqueAAs <- UniqueAAs[apply(UniqueAAs,1,function(row) all(row !=
 0)),]
UniqueAAs <- na.omit(UniqueAAs)
UniqueAAsR3 <- UniqueAAs

#this counts sequences that have TAG codons, sequences that have more
 than one are only counted once
TAGreg <- regexpr("\\sTAG",codons)
TAGtable <- table(TAGreg)
percentTAG <-
 sum(TAGtable[2:length(TAGtable)])/length(codons)*100#percent of
 sequences containing TAG

#this creates a matrix of amino acid sequences that do not contain TAG
 codons
TAGpos <- which(AAs == "TAG")
TAGrow <- TAGpos%%nrow(AAs)
AAsnoTAG <- AAs[-TAGrow,]
```

```
#this creates heatmap for amino acid frequency per library position,
 change scale according to values
AAtable <- apply(AAs,2,function(x) table(factor(x,levels=aa)))
AAtable <- as.matrix(AAtable/length(codons))
colnames(AAtable) <- c(1:lib)
heatmapcolors <- colorRampPalette(brewer.pal(9,"Blues"))(100)
sc <- seq(0.0,0.6,by=0.006)
AAheatmap <- heatmap.2(AAtable, Rowv = NA, Colv = NA, col =
 heatmapcolors, density.info = "none", scale = "none", trace = "none",
 breaks = sc,  xlab = "Position in Library", ylab = "Codon", margins =
 c(3,4), dendrogram = "none")

#this creates projected heatmap based on NNK randomized codons
randomAAs <- matrix(0,21,lib,dimnames =
 list(rownames(AAtable),c(1:lib)))
randomAAs[c("A","G","P","T","V"),] <- 2/32
randomAAs[c("C","H","Q","N","K","Y","D","E","W","I","M","TAG","F"),]
 <- 1/32
randomAAs[c("L","S","R"),] <- 3/32
NNKheatmap <- heatmap.2(randomAAs, Rowv = NA, Colv = NA, col =
 heatmapcolors, density.info = "none", scale = "none", trace = "none",
 breaks = sc,  xlab = "Position in Library", ylab = "Codon", margins =
 c(3,4), dendrogram = "none")

#this creates heatmap showing bias from random, change scale with
 respect to range of values
lscale <- seq(-1,4,by=5/100)
librarybias <- (AAtable - randomAAs)/randomAAs
Biasheatmap <- heatmap.2(librarybias, Rowv = NA, Colv = NA, col =
 heatmapcolors, density.info = "none", scale = "none", trace = "none",
 breaks = lscale,  xlab = "Position in Library", ylab = "Codon",
 margins = c(3,4), dendrogram = "none")
librarybias <- as.data.frame(librarybias)

#this creates heatmap for AAsnoTAG
AAnoTAGtable <- apply(AAsnoTAG,2,function(x)
 table(factor(x,levels=aa)))
AAnoTAGtable <- as.matrix(AAnoTAGtable/nrow(AAsnoTAG))
colnames(AAnoTAGtable) <- c(1:lib)
heatmapcolors <- colorRampPalette(brewer.pal(9,"Blues"))(100)
sc <- seq(0.0,0.3,by=0.003)
AAheatmap <- heatmap.2(AAnoTAGtable, Rowv = NA, Colv = NA, col =
 heatmapcolors, density.info = "none", scale = "none", trace = "none",
```

```
breaks = sc,  xlab = "Position in Library", ylab = "Codon", margins =
c(3,4), dendrogram = "none")

#writes csv files for uniqueAAs and bias heatmaps change path to make
 file
path <- "/Users/traehampton/Documents/Research/Sequencing Results/Next
 Gen Sequencing/21510Wns_N21170"
write.csv(UniqueAAs, paste(path,"/12mernegnegUniqueAAs.csv", sep =
 ""), row.names = F)
write.csv(TAGtable, paste(path,"/12mernegnegTAGtable.csv", sep = ""),
 row.names = T)
write.csv(librarybias, paste(path,"/12mernegnegLibraryBias.csv", sep =
""), row.names = T)
```

Supplementary Script 2: Script for Enrichment Analysis

```
#this gives overall enrichment of each peptide sequence by fold change
 between R1 and R4
library(prodlim)
path <- "/Users/traehampton/Documents/Research/Sequencing Results/Next
 Gen Sequencing/23011Wns_N23003"
lib <- 14
match <-
 row.match(as.data.frame(UniqueAAs27R1[,1:lib]),as.data.frame(UniqueAAs
 27R4[,1:lib]))
matchseq <- which(is.na(match)==FALSE)
log2foldchange <-
 log2((UniqueAAs27R4[match[matchseq],lib+1]/sum(UniqueAAs27R4[,lib+1]))
 /(UniqueAAs27R1[matchseq,lib+1]/sum(UniqueAAs27R1$n)))
enrichedseq <- UniqueAAs27R4[match[matchseq],]
enrichedseq$log2foldchange <- log2foldchange[,1]
enrichedseq <- enrichedseq[order(-enrichedseq$log2foldchange),]
enrichedseq <- as.data.frame(enrichedseq)
UniqueAAs27R1$percent <- UniqueAAs27R1$n/sum(UniqueAAs27R1$n)*100
UniqueAAs27R4$percent <- UniqueAAs27R4$n/sum(UniqueAAs27R4$n)*100
#Change names to whatever you want for each data frame
write.csv(enrichedseq, paste(path,"/27R1vR4.csv", sep = ""), row.names
 = F)
write.csv(UniqueAAs27R1, paste(path,"/28R1.csv", sep = ""), row.names
 = F)
write.csv(UniqueAAs27R1, paste(path,"/28R2.csv", sep = ""), row.names
 = F)
write.csv(UniqueAAs28R3, paste(path,"/28R3.csv", sep = ""), row.names
 = F)
```

```
write.csv(UniqueAAs27R4, paste(path,"/28R4.csv", sep = ""), row.names =
 F)
```
